# The Effects of Lengths of Flavin Surfactant *N*-10-Alkyl Side Chains on Promoting Dispersion of a High-Purity and Diameter-Selective Single-Walled Nanotube

**DOI:** 10.3390/nano12193380

**Published:** 2022-09-27

**Authors:** Minsuk Park, Seongjoo Hwang, Sang-Yong Ju

**Affiliations:** Department of Chemistry, Yonsei University, 50 Yonsei-ro, Seodaemun-gu, Seoul 03722, Korea

**Keywords:** single-walled carbon nanotube, dispersibility, flavin, side chain length, tandem, diameter selectivity

## Abstract

Flavin with defined helical self-assembly helps to understand chemical designs for obtaining high-purity semiconducting (*s*)-single-walled carbon nanotubes (SWNT) in a diameter (*d*_t_)-selective manner for high-end applications. In this study, flavins containing 8, 12, 16, and 20 *n*-alkyl chains were synthesized, and their single/tandem effects on *d*_t_-selective *s*-SWNT dispersibility were investigated at isomolarity. Flavins with *n*-dodecyl and longer chain lengths (FC12, FC16, and FC20) act as good surfactants for stable SWNT dispersions whereas *n*-octyl flavin (FC8) exhibits poor dispersibility owing to the lack of SWNT buoyancy. When used with small-*d*_t_ SWNT, FC8 displays chirality-selective SWNT dispersion. This behavior, along with various flavin helical motifs, prompts the development of criteria for ‘side chain length (*l*_S_)’ required for stable and *d*_t_-selective SWNT dispersion, which also explains *l*_S_-dependent *d*_t_-enrichment behavior. Moreover, SWNT dispersions with flavins with dodecyl and longer *l*_S_ exhibit increased metallic (*m*)-SWNT, background absorption-contributing carbonaceous impurities (CIs) and preferential selectivity of *s*-SWNT with slightly larger *d*_t_. The increased CIs that affect the SWNT quantum yield were attributed to a solubility parameter. Furthermore, the effects of flavin *l*_S_, sonication bath temperature, centrifugal speed, and surfactant concentration on SWNT purity and *s*-/*m*-SWNT ratio were investigated. A tandem FC8/FC12 provides fine-tuning of *d*_t_-selective SWNT dispersion, wherein the FC8 ratio governs the tendency towards smaller *d*_t_. Kinetic and thermodynamic assemblies of tandem flavins result in different sorting behaviors in which wide *d*_t_-tunability was demonstrated using kinetic assembly. This study highlights the importance of appropriate side chain length and other extrinsic parameters to obtain *d*_t_-selective or high-purity *s*-SWNT.

## 1. Introduction

Single-walled carbon nanotubes (SWNTs), hollow cylindrical forms of rolled-up graphene, are an excellent candidate for next-generation nanodevices. SWNTs have attracted significant attention as next-generation thin-film transistors and energy materials owing to their lightweight and excellent electrical [1,2,3,4] and thermal [5,6,7] properties. However, in these applications, SWNTs in mixtures require separation into metallic (*m*) and semiconducting (*s*) forms. Well-known *s*-SWNT separation methods include ion-column chromatography using DNA [8,9,10], density gradient centrifugation [11,12,13], gel chromatography [14,15,16], aqueous two-phase extraction [17,18,19], and dispersions using poly(fluorene) (PFO) [20,21,22] or flavins [23,24,25,26]. Among these approaches, PFO or flavin derivatives as dispersants enable *s*-enriched SWNT dispersions by one-pot sonochemical dispersion [20,21,24,25].

**Figure 1:**
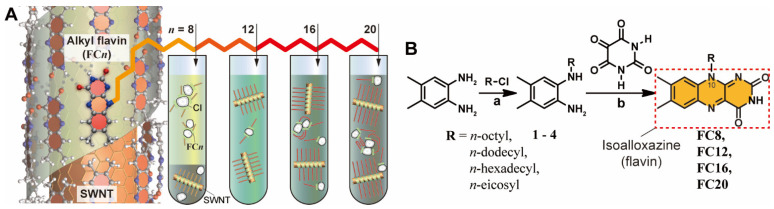
(**A**) Schematic showing the effect of flavin *l*_S_ on the SWNT dispersions. (Left) SWNT wrapped by helical isoalloxazine wrapping with *n*-alkyl side chains. (Right) *l*_S_-induced SWNT dispersion containing flavin-wrapped SWNTs and CIs with different degree. (**B**) Synthetic routes to prepare **FC8**, **FC12**, **FC16**, and **FC20**. Note that N10 site for different *l*_S_ is indicated. Conditions: (a) TEA, 130 °C, 12 h; yield: **1**: 32%, **2**: 60%, **3**: 46%, **4**: 25%; (b) glacial acetic acid, B_2_O_3_, 60 °C, 6 h; yield; **FC8**: 56%, **FC12**: 58%, **FC16**: 53%, **FC20**: 50%.

Flavins possess a well-defined self-assembly motif for dispersing carbon nanotubes [27,28,29,30,31] and could be easily removed [25] for high-end applications. As shown in Figure 1A, the isoalloxazine ring of the flavin wrapping system exhibits a strong π–π interaction with SWNT and quadruple hydrogen-bonding with the facing isoalloxazine, and its side chain provides dispersibility [27,28]. Flavin mononucleotide (FMN) and *n*-dodecyl flavin (**FC12**), which exhibit varied side chains, have been demonstrated as efficient dispersing agents in water [27,32] and organic solvents [24,28,33]. In this regard, efforts have been made to assess how the changes in the flavin core structure, which participates in direct π–π interactions with SWNT, affect the SWNT separation and resultant dispersibility [25]. Particularly, flavins with *n*-octadecyl side chains have larger SWNT diameter (*d*_t_) selectivity than those with dodecyl chains [25]. In addition, PFO with longer side chain length (*l*_S_) results in the selection of larger *d*_t_ SWNT owing to the side chain interaction with SWNT [34]. In the PFO system, one of the dual alkyl side chains in PFO acts as an SWNT-interacting group, selecting different *d*_t_ by change the distance between surfactants, whereas the other provides dispersibility [34]. Moreover, polythiophene exhibits a similar larger-*d*_t_ selectivity with increasing *l*_S_ [35,36,37]. Because flavin side chain does not directly interact with the SWNT sidewalls, its distinct role compared to that of PFO can provide insight into designing novel surfactants with adequate length. Therefore, it is understood that surfactants with specific *l*_S_ may be selective for smaller-*d*_t_ SWNT, which requires verification.

Surfactant has specific binding affinity with SWNT metallicity, *d*_t_, and handedness. Therefore, different surfactant concentrations lead to different SWNT species selection. When comparing surfactants with analogous SWNT-interacting cores and different *l*_S_, it is imperative to reveal the effect of *l*_S_ while excluding concentration-driven SWNT selectivity. In this regard, we reported that a surface stoichiometry between the flavin footprint/surface area of SWNT exists, and the surfactant/SWNT ratio plays an important role in selecting SWNT chiralities [33,38]. Therefore, the effects of *l*_S_ in equimolar flavin can explicitly reveal the relationship between SWNT dispersibility and selectivity for obtaining high-purity *s*-SWNT. Furthermore, the simultaneous use of flavins with different *l*_S_ further fine-tunes *d*_t_ selectivity and has not been explored yet.

In this study, we synthesized four flavin derivatives and investigated their single/tandem effects on *d*_t_-selective dispersion of high-purity *s*-SWNT in equimolar flavin. First, the flavin derivatives with octyl to eicosyl *l*_S_ (that is, **FC8**, **FC12**, **FC16**, and **FC20**) were synthesized and characterized using various techniques including nuclear magnetic resonance (NMR) and elemental analysis. Notably, increasing *l*_S_ from **FC12** to **FC20** increased the *m*-SWNT and carbonaceous impurities (CIs), whereas **FC8** exhibited poor dispersion for larger *d*_t_ SWNT and chirality-selective dispersion for smaller *d*_t_ SWNT. The *l*_S_ per carbon atom in SWNT for providing marginal/good SWNT dispersibility was derived using *d*_t_-selective dispersion. The increased CI content in the SWNT dispersion with increasing *l*_S_ was ascribed to the matched solubility parameter (*δ*). Various parameters such as *l*_S_, centrifugal force, sonication bath temperature, and surfactant concentration were investigated to control the amounts of *s*-/*m*-SWNT and SWNT purity. Finally, the tandem use of **FC8**/**FC12** to disperse and control the distribution of SWNT was investigated. The combination of poor and good surfactants results in a large tunability of the selected *d*_t_ range. Initiated by different **FC8**/**FC12** ratios on SWNT for as-prepared and aged samples, social or narcissistic sortings of flavin self-assembly were demonstrated for sonication- and aging-assisted dispersions.

## 2. Materials and Methods

### 2.1. Materials and Instrumentation

Synthesis of flavin derivatives and precursors followed the previous literature [28], which will be elaborated in the Supplementary Materials. 4,5-Dimethyl-1,2-phenylenediamine, 1-chlorooctane, 1-chlorododecane, 1-chlorohexadecane, and 1-chloroeicosane were purchased from TCI. All solvents and chemicals were reagent grade and were used as-received. As-purchased plasma-grown SWNT (PSWNT) (RN-220 SWCNTs, batch #: R26-036, nanotube purity of 30–70%, Nano Integris Inc., Boisbriand, QC, Canada) was used. The SWNT *d*_t_ distribution was 1.3 ± 0.35 nm. High-pressure carbon monoxide (HiPco) process SWNT (*d*_t_ distribution: 1.0 ± 0.35 nm, Nano Integris Inc., Boisbriand, QC, Canada) [39] was also used for control. For synthesis, silica gel with 70–230 mesh (Silica gel 60, Merck, Darmstadt, Germany) was used as a stationary phase for flash chromatography. All measurements were carried out at room temperature unless otherwise noted. Melting points (m.p.) of the synthesized compounds were measured using a m.p. apparatus (SMP3, Stuart Scientific, St Neots, UK). ^1^H and ^13^C NMR spectra were acquired on a FT-NMR spectrometer (Biospin Avance II and Avance III HD 400, Bruker, Billerica, MA, USA) operating at Larmor frequencies of 400 MHz and 100 MHz, respectively. All spectra were recorded in 5-mm NMR tubes containing 0.70 mL of CDCl_3_ (DLM-7TB-100S, Cambridge Isotope Lab., Tewksbury, MA, USA) with 0.05% tetramethylsilane (TMS) as an internal reference at 295 K. Elemental analysis was conducted using an elemental analyzer (2400 CHNS/O series II, PerkinElmer, Waltham, MA, USA) in the CHN acquisition mode and calibrated against acetanilide as a reference with a typical error range of ±0.2%. Solubility of flavins in *p*-xylene was obtained by absorption measurement. Those absorption spectra were obtained from the solution by heating excess flavins in *p*-xylene and subsequent centrifugation (5000 *g* for 10 min) and filtration (poly(vinylidene fluoride), 0.2 μm). The resulting solubility was obtained by using extinction coefficient (12,600 L/mol·cm) [40,41]. Fourier-transform infrared (FTIR) spectra were acquired from JASCO FT/IR 4700 (Tokyo, Japan) by using pellets from ground sample/KBr.

### 2.2. Preparation of SWNT Dispersion

A mixture of PSWNT (1 mg) and flavin derivatives (0.61 mM, i.e., 0.86, 1.00, 1.13, and 1.27 mg for respective **FC8**, **FC12**, **FC16**, and **FC20**, respectively) was added to *p*-xylene (4 mL) which was dried over 3 Å molecular sieve (Alfa Aesar, Haverhill, MA, USA) overnight before use. The resulting mixture was subjected to 5 min bath sonication (Branson 1510, 70 W, Emerson, Saint Louis, MO, USA) for mixing, and further to 1 h tip sonication (40% power, 18.8 W/mL, probe tip diameter: 13 mm, VCX 750, Sonics & Materials, Newtown, CT, USA). During sonication, the temperatures of the sample vials were maintained at 15, 25, and 35 °C using an external water circulator (Lab Companion RW-2025G, Jeio Tech, Daejeon, Korea). Centrifugations with typical (5 and 30 k*g*) and optional (1 and 60 k*g*) *g* forces were conducted by using a high-performance centrifuge with a fixed angle rotor (Avanti J-26 XPI and JA-25.50, respectively, Beckman Coulter, Brea, CA, USA) at room temperature using an organic solvent-tolerant centrifugal tube (50 mL, Cat. #: 3114-0050, fluorinated poly(ethylene-*co*-propylene), Nalgene, Sigma-Aldrich, Saint Louis, MO, USA). 80% of the supernatant was carefully collected for further measurements. For dispersions with lower and higher flavin concentrations, 0.5 and 2.44 mM were utilized. HiPco SWNT was dispersed in a similar manner as mentioned above.

#### Tandem Surfactant Dispersion

SWNT dispersions with tandem surfactants were obtained by mixing **FC12** and other flavin derivatives (i.e., **FC8**, **FC16**, and **FC20**) to maintain 0.61 mM with a similar protocol as mentioned above. Aging experiments were conducted at room temperature.

### 2.3. Absorption Measurement

UV-vis-short-wavelength infrared (SWIR) absorption spectra were recorded on a JASCO V-770 (Tokyo, Japan) with cuvettes having 1 mm path length (21/Q/1, Starna scientific, Ilford, UK) unless otherwise noted. SWIR rather than near IR was utilized because of the extended measurement up to 2100 nm. Absorbances were measured via a double-beam configuration. Wavelength accuracies for UV-vis and for IR regions were 0.3 nm and 1.5 nm, respectively.

### 2.4. Photoluminescence Excitation (PLE) Measurement

The measurements were conducted using a Spex Nanolog 3-211 spectrofluorometer (Jobin Yvon, Horiba, Japan). A 450 W xenon lamp (220–1000 nm, Ushio, Tokyo, Japan) was utilized as a light source, according to the literature [32]. A double monochromator with 1200 g/mm blazed at 500 nm was utilized for wavelength selection. Emission from a sample was collected from 90° of the excitation direction, filtered by a 830-nm long-pass filter (RG-830, Schott, Mainz, Germany) and entered into spectrometer (iHR320, 150 g/mm, Horiba, Kyoto, Japan) equipped with a liquid N_2_-cooled InGaAs array detector (512 × 1 pixels, Symphony II IGA 1600, Horiba, Kyoto, Japan) for the near infrared (NIR) range. The excitation and emission intensities were corrected against instrumental variations using sensitivity correction factors. For PLE map measurement of SWNT, the bandwidth of excitation and emission wavelengths were 14 nm and 10 nm, respectively. The increments of the excitation and emission wavelengths were 5 and 1 nm, respectively. Fluorescence quartz cuvette (QS, Hellma, Plainview, NY, USA) with a 10 mm beam-pass was utilized for the measurement. The InGaAs detector in our setup had a detection limits up to 1600 nm.

### 2.5. Atomic Force Microscopy (AFM) Measurement

The measurements were conducted by using a commercially available AFM (NX10, Park Systems, Suwon, Korea). An Al-coated silicon cantilever with a spring constant 37 N/m, a resonance frequency of 300 kHz and a quoted radius of approximately 6 nm (ACTA, App Nano, Mountain View, CA, USA) were utilized to measure height topographies. A 512 × 512 pixel image was collected from a 5 μm × 5 μm area. Prior to AFM measurement, 200 μL of the PSWNT dispersion was dropcast on 285 nm-thick SiO_2_/Si or mica substrates and dried for 10 min. For flavin-wrapped SWNT, the sample was dropcast on a freshly cleaved mica and was rinsed with *p*-xylene several times to preserve flavin self-assembly on SWNT while extra flavins were removed. SWNT images were acquired at a resolution of 1024 × 1024 pixels from a 1 μm × 1 μm area. For the case of flavin-removed SWNT, the flavins/SWNT on SiO_2_/Si substrate was rinsed with acetone several times until no green fluorescence originating from flavin derivatives was observed upon irradiation with a hand-held UV lamp (i.e., 365 nm). The rinsed substrate was dried by a gentle N_2_ blow. The measured height and phase topographies were analyzed by using the XEI 4.3.4 program. The height distribution of CIs was extracted from more than four topographies per sample in which approximately 4600 and 3000 CIs (i.e., 5 and 30 kg dispersion, respectively) were analyzed.

### 2.6. Raman Measurement

The measurements were performed by using a custom-made micro Raman setup [42]. via back scattering geometry. Briefly, scattered light from two different lasers (785 and 532 nm, 0785-08-11 and 08-DPL 532 nm 100 mW; Cobolt AB, Solna, Sweden) was collected via 50× objective lens (MPlan, N.A.: 0.75, Olympus, Japan) and delivered to spectrometer with a Si array CCD (Triax 320, focal length: 320 mm, 1800 gr/mm, resolution: 2 cm^−1^; 26 μm/pixel, 1024 × 256, Syncerity; Horiba Jobin-Yvon, Kyoto, Japan). The laser spot size is approximately 1 μm and an additional Si peak at 520.89 cm^−1^ was used as an internal reference. A total of 50 μL of SWNT dispersion was dropcast on a 285 nm-thick SiO_2_/Si substrate and dried at 90 °C on a hot plate. The sample was concentrated by repeating this process several times and washed with copious amounts of acetone to remove flavins. The laser power was below 0.2 mW to minimize sample damage. The spectra were acquired by averaging several points and were normalized against the maximum at each region. For Raman spectra of flavin derivatives, 785-nm excitation on powder sample was utilized with power of 50 mW, whereas 532-nm excitation results in fluorescence emission of flavin derivatives.

### 2.7. Geometrical Modeling of Flavin-Wrapped SWNT

Visualization of flavin wrapping on SWNT and strained graphene: *xyz* coordinates of various SWNTs were generated by using free software based on C–C distance of 1.42 Å [43]. Flavin dimers and subsequent *8*_1_ flavin helix on (6,8) tube was generated according to the method in the literature [27]. More vigorous building methods are listed in Supplementary Materials. Molecular mechanics simulation was performed by using COMPASS force field with convergence tolerance of 0.9 μeV for geometry optimization implemented in Materials Studio. Hexagonal unit cell containing three unit lengths of (8,10) and four unit lengths of *9*_1_ flavins is utilized for the initial configuration. Side chain was omitted for clarity. Visual molecular dynamics (VMD) software was used for molecular visualization [44].

### 2.8. Transmission Electron Microscopy (TEM) Measurement

The measurements were conducted by using Tecnai G^2^ F30ST (FEI company, Hillsboro, OR, USA) operating at 300 kV acceleration voltage. For TEM measurement, 1 mg of as-purchased PSWNT was mixed with 4 mL *N*-methyl-2-pyrrolidone (NMP) and subjected to 10 min bath sonication. Subsequently, TEM grids covered with ultrathin carbon support (LC200-Cu, lot no.: 180912, 200 mesh, TED Pella, Redding, CA, USA) were floated upside-down on top of a drop of the prepared PSWNT dispersion on a slide glass substrate. Excess sample was washed with NMP. Then, the grid was dried under vacuum overnight.

### 2.9. Scanning Electron Microscopy (SEM) Measurement

The measurements were conducted by using Schottky emission SEM (SU-70, Hitachi, Tokyo, Japan) operating at 10 kV acceleration voltage. To prepare SWNT film for SEM measurement, SWNT dispersions (10 mL) were filtered using a 1.0 μm pore-sized poly(tetrafluoroethylene) (PTFE) filter (PTFE1025D, Hyundai Micro, Seoul, Korea) and washed with acetone to remove residual flavin. Images of SWNT films were typically obtained with a working distance of ca. 11 mm under 100 k magnification. All samples were measured without conductive metal coating.

### 2.10. Matrix-Assisted Laser Desorption Ionization–Time of Flight (MALDI-TOF) Mass Spectrometry (MS) Measurement

#### 2.10.1. Sample Preparation

To determine the composition of tandem flavin derivatives on the SWNT surface, flavin-adsorbed SWNT was selectively collected by filtration method. SWNT dispersions with tandem surfactants denoted by the as-prepared and the aged samples were filtered by PTFE membrane filter (pore size: 0.1 μm, J010A025A, Advantec, Tokyo, Japan). SWNT dispersion (10–20 μL) was carefully dropped repeatedly to the same spot (spot size: <2 mm) by using a micropipette, which was rinsed with *p*-xylene (few mL) to remove excess flavin on the filter. A piece of filter containing SWNT was placed into an eppendorf tube with 50 μL of *p*-xylene and was subjected to bath sonication for redispersion of SWNT. For the measurement, 2 μL sample dispersion was mixed with 2 μL of 88.4 mM dithranol in tetrahydrofuran as matrix, and 2 μL of the mixed solution was dropcast on the MSP 96 target plate (Bruker, Billerica, MA, USA).

#### 2.10.2. Measurement

The measurement was performed on a Autoflex Max (Bruker, Billerica, MA, USA) equipped with smartbeam-II solid-state pulsed laser (wavelength: 355 nm, laser spot size: <10 μm, repetition rate: 2 kHz). Laser power was set to 40% of the maximum peak intensity (>100 μJ/pulse). Positive ions were detected in the reflection mode. A total of 20,000 laser shots were applied to the sample. The composition ratio of **FC8** and **FC12** was determined by comparing MS spectrum regions at specific mass to charge ratio (*m*/*z*) from **FC8** and **FC12**.

## 3. Results

In this study, we investigated the single/tandem effects of flavin *l*_S_ on the dispersion of *s*-SWNT, as shown schematically in Figure 1A. SWNT is helically wrapped by the flavin or isoalloxazine moiety, and its side tail extends to the media. A single SWNT batch contains not only SWNT but also CIs and a metallic catalyst. Although the metallic catalyst can be removed by centrifugation [45], the CIs, which are either amorphous and graphitic forms of carbon [46,47] or defective few-layered graphene material [48] depending on SWNT batches, are inevitably incorporated into the dispersion owing to structural similarities with SWNT. To assess the effects of alkyl *l*_S_ in flavin surfactants on SWNT dispersion, we synthesized four flavin derivatives, viz. **FC8**, **FC12**, **FC16**, and **FC20** (Figure 1B), containing N10-linked 8, 12, 16, and 20 *n*-alkyl side chains, respectively. The syntheses were performed over two steps, involving the mono-alkylation of 4,5-dimethyl-1,2-phenylenediamine followed by the construction of an isoalloxazine ring according to a literature-reported protocol [24,28]. Briefly, reactions between three equivalents of 4,5-dimethyl-1,2-phenylenediamine and one equivalent of 1-chlorinated octane, dodecane, hexadecane, and eicosane were performed in refluxing TEA to form the respective monoalkylated phenylenediamine derivatives **1**–**4** (yield: 25–60%). Thereafter, reactions between **1**–**4** and alloxan in a 1:1 molar ratio were performed in glacial acetic acid in the presence of boric oxide to produce the corresponding flavin derivatives **FC8**, **FC12**, **FC16**, and **FC20** (yield: 50–58%).

The structures of the *n*-alkyl flavins and intermediates **1**–**4** were confirmed using elemental analysis, ^1^H-NMR (Figures S1A–D and S3A–D in the Supplementary Materials, respectively), and ^13^C-NMR spectroscopies (Figures S2A–D and S4A–D, respectively) (see Supplementary Materials). The signals in the ^1^H NMR spectra of **FC8**, **FC12**, **FC16**, and **FC20**, and **1**–**4** were identified as per previous literature [25,28,49]. For instance, as shown in the ^1^H-NMR spectra of all flavin derivatives (Figure S3A–D), the positions and intensities of the aromatic protons above 7 ppm remained unchanged, whereas the intensities of the aliphatic side chain protons at 1.3 ppm increased with increasing length of the flavin side chain. Consequently, these flavin derivatives were utilized to disperse SWNT.

SWNT dispersions were prepared in *p*-xylene by using sonication in the presence of flavin derivatives as surfactants, as per literature [24,28,33,48]. In particular, equimolar flavin derivative (0.61 mM) was added to PSWNT (0.25 wt./v.%) with a *d*_t_ value ranging from 0.95 to 1.65 nm in *p*-xylene to eliminate the concentration-dependent effect. After dispersion through tip sonication and subsequent centrifugation at 5 or 30 k*g* (see Materials and Methods section for the details), 80% of the supernatants were obtained using **FC12**, **FC16**, and **FC20**. Unless otherwise stated, the dispersion derived from **FC8** was not centrifuged owing to the instability of SWNT dispersion.

**Figure 2:**
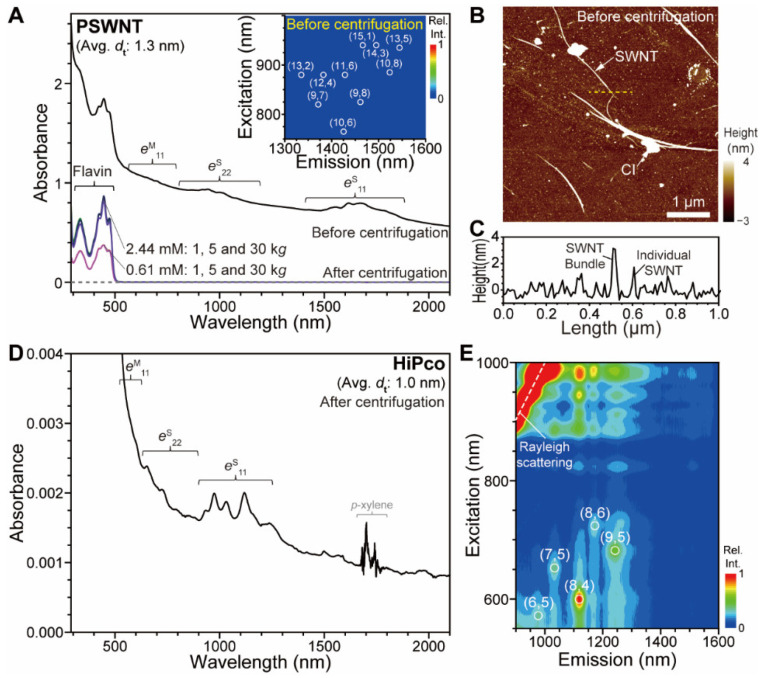
*d*_t_-dependent **FC8**-SWNT dispersions. (**A**) UV-vis-SWIR absorption spectra of **FC8**-PSWNT dispersions with or without centrifugation. Inset: PLE map of **FC8**-PSWNT dispersion before centrifugation. (*n*, *m*) and circle denote the SWNT chiralities and their cross position between *e*^S^_22_ and *e*^S^_11_ according to the literature [24]. (**B**) AFM height image of **FC8**-PSWNT dispersion without centrifugation deposited on 285 nm-thick SiO_2_/Si substrate. (**C**) Line profile of the dashed line in (**B**). (**D**) Absorption spectrum of 5-k*g*-centrifuged 2.44 mM **FC8**-HiPco dispersion, acquired by using a cuvette of beam path 10 mm. The feature near 1650–1800 nm was originated from overtones of C–H stretching of *p*-xylene [50]. (**E**) The corresponding PLE map showing smaller *d*_t_ enrichment of SWNT.

**FC8**-PSWNT dispersion does not show good PSWNT dispersibility compared to other flavin derivatives, and exhibits *d*_t_-selective SWNT dispersion when smaller-*d*_t_ SWNT is used. Figure 2A shows UV-vis-SWIR absorption spectra of **FC8**-PSWNT with and without the 5- and 30-k*g* centrifugation step. Without centrifugation, the dispersion exhibits broad background absorption and broad optical transitions, suggesting a bundled SWNT structure. Specifically, the absorption spectrum contains the first and second semiconducting excitonic transitions (*e*^S^_11_ and *e*^S^_22_, respectively) of the PSWNT in the wavelength ranges of 800–1200 nm and 1200–2100 nm, respectively, which are in good agreement with previous literature [24]. Moreover, weak absorption bands originating from the first metallic excitonic transitions (*e*^M^_11_) are observed in the wavelength range of 600–800 nm. The PLE map that determines optical transitions of *s*-SWNT (inset of Figure 2A) does not show photoluminescence (PL) activity originating from SWNT chiralities denoted by (*n*, *m*), indicating that the dispersion mostly consists of bundled SWNT. Progressively decreasing centrifugal forces (30, 5, and 1 k*g*, bottom spectra of Figure 2A) and increasing flavin concentration to 2.44 mM still result in large amounts of PSWNT precipitation, as is evident from absence of the SWNT absorption, except in the flavin absorption region (300–500 nm). This result suggests that **FC8** shows poor PSWNT dispersibility. AFM was utilized to investigate the detailed SWNT topography. Figure 2B clearly shows that most SWNT exist as bundled states and only a few as individualized states. The height profile (Figure 2C) indicates that bundled and individualized SWNT exhibit heights greater than 3.5 nm and 2.0 nm, respectively. Considering PSWNT diameter up to 1.65 nm, this result suggests that while the flavin moiety wraps around PSWNT, its tail length is insufficient to provide SWNT buoyancy. To demonstrate this point, HiPco SWNT, whose average *d*_t_ range (0.65–1.35 nm) is smaller than that of PSWNT, was subjected to **FC8** dispersion with 5-k*g* centrifugation in a similar manner as that for PSWNT. While the 0.61 mM dispersion is unstable, the 2.44 mM **FC8** dispersion exhibits sharp absorption features originating from HiPco SWNT, as shown in Figure 2D. Interestingly, the corresponding PLE map (Figure 2E) exhibits smaller-*d*_t_ chirality enrichments of SWNT in the order (8,4), (9,5), (8,6), (7,5), and (6,5) tubes. Their average *d*_t_ is 0.86 nm, which contrasts sharply with that of the 2.44 mM **FC12**-HiPco dispersion: The absorption results (Figure S5A) show much larger *d*_t_ distribution, with *e*^S^_11_ extending up to 1600 nm, and the corresponding PLE map (Figure S5B) shows that PL-based chiralities range from (6,5) to (10,8) with a number-average *d*_t_ of 0.96 nm. PL-intensity based chirality abundances of **FC12**- and **FC8**-HiPco were illustrated as Weisman plots in Figure S5C. Clearly, **FC8**-HiPco exhibits smaller *d*_t_ enrichment whose most abundant species are (8,4) as 38% as compared to **FC12**-HiPco in which comparably larger-*d*_t_ (9,5), (8,6), and (7,6) altogether accounts for 32%. These results indicate that *d*_t_-selective SWNT dispersion originates from *l*_S_.

**Figure 3:**
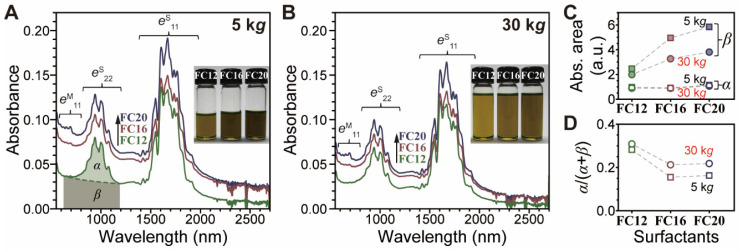
SWNT dispersibility of flavin derivatives as function of increasing *l*_S_ and varying centrifugation force. UV-vis-SWIR absorption spectra of PSWNT dispersions by using **FC12** (olive), **FC16** (brown), **FC20** (navy) and centrifugal forces of (**A**) 5 and (**B**) 30 k*g*. *α* and *β* denote SWNT contribution and background absorption, respectively. The noise signals in the region from 2500 to 2700 nm originates from infrared vibrations of residual water [48]. Insets display photographs of each set of the dispersions, showing tone-down of the dispersions with 30 k*g*. (**C**) *α* (empty), *β* (solid), and (**D**) *α*/(*α* + *β*) contributions according to centrifugal forces (i.e., 5 k*g* (square) and 30 k*g* (circle)) and *l*_S_.

The remaining flavin surfactants exhibit preferential *s*-SWNT enrichment and flavins with longer *l*_S_ exhibit slightly larger *d*_t_ selectivity. Figure 3A,B shows the absorption spectra of PSWNT dispersions obtained using **FC12**, **FC16**, and **FC20** with respective centrifugations. In contrast to **FC8**, the absorption spectra of 5 k*g* of centrifuged dispersions obtained using **FC12**, **FC16**, and **FC20** contain well-resolved *e*^S^_11_ and *e*^S^_22_ transitions. While the indicated *s*-SWNT absorption, including *e*^S^_11_ and *e*^S^_22_, does not appear to change significantly, weak absorption bands originating from *e*^M^_11_ are present in the 600–800 nm range and increases with increasing *l*_S_. Additionally, this trend is accompanied by an increased background absorption. However, a closer inspection of background-subtracted absorption spectra (Figure S6) reveals that flavins with longer *l*_S_ tend to have larger *d*_t_. This result is consistent with the findings that the *l*_S_ variation of PFO [20,34] and flavin derivatives [25] affects the SWNT chirality selection owing to the side chain involvement for SWNT wrapping. Because of the quadruple hydrogen-bonding of flavin helix, the side chain of flavin is not expected to make contact with SWNT sidewalls [27,28], and these effects (i.e., inclusion of a small amount of *m*-SWNT) appear to originate from the increase in dispersibility caused by increasing side chain. To investigate this, PSWNT dispersions using lower flavin concentrations (i.e., 0.5 mM) were conducted in a similar protocol. The resulting absorption spectra (Figure S7A,B) show that, as opposed to 0.61 mM dispersions, these dispersions exhibit a near absence of *m*-PSWNT irrespective of *l*_S_ while still exhibiting lower background absorption. Because N10-alkyl flavin preferentially selects *s*-SWNT [24], this result clearly indicates that flavin dispersibility and stoichiometry between flavin and SWNT are crucial for better selection of *s*-SWNT, in accordance with chirality selectivity by flavin concentration change [33]. In this regard, the shorter the *l*_S_ of **FC12** than those of **FC16** and **FC20,** the better is the *s*-SWNT enrichment.

Second, background absorptions increase with increasing *l_S_*. This result indicates that a longer side chain increases the dispersibility of both SWNT and background absorption. Especially in aromatic solvents, the background absorption in flavin/SWNT dispersion mainly originates from CIs [33]. CIs are few-layered defective graphene material, which contributes to background absorption along with the SWNT bundle [46,47]. For a qualitative comparison of this effect, the background absorption (*β*) and background-subtracted SWNT absorbance (*α*), based on the *e*^S^_22_ and *e*^M^_11_ region on the *x* axis in wavenumber scale, are defined as shown in Figure S8A–F, according to the literature [51] wherein the *e*^S^_22_ enclosure was drawn from 8400 to 16,000 cm^−1^ (i.e., 1190–625 nm). In this range, *α* is further classified into *s*-SWNT (*α_sem_*) and *m*-SWNT (*α_met_*). Evidently, the increasing side chain and reduced centrifugation results in larger *β*. Figure 3C,D show the trends of *α*, *β* and *α*/(*α* + *β*) as figures of merit for determining the purity of the dispersions. With increasing *l*_S_, *α* increases slightly owing to an increase in the *m*-SWNT content, whereas *β* increases drastically. This result clearly suggests that longer *l*_S_ incorporates more CIs. Moreover, *α*/(*α* + *β*), which denotes net SWNT contribution among SWNT and CIs, decreases with increasing *l*_S_ (Figure 3D). The best *α*/(*α* + *β*) value was obtained from **FC12**-PSWNT with a 30-k*g* centrifugation, resulting in a value of 0.309, which is slightly lower (i.e., 0.403) than that of PFO-SWNT dispersion [51]. Alternatively, an increased centrifugal force results in reduced *β*, as shown Figure 3C. Notably, dispersions obtained from 60-k*g* centrifugation (Figure S9A–C) afford *α* and *β* values similar to those of the 30-k*g* samples.

**Figure 4:**
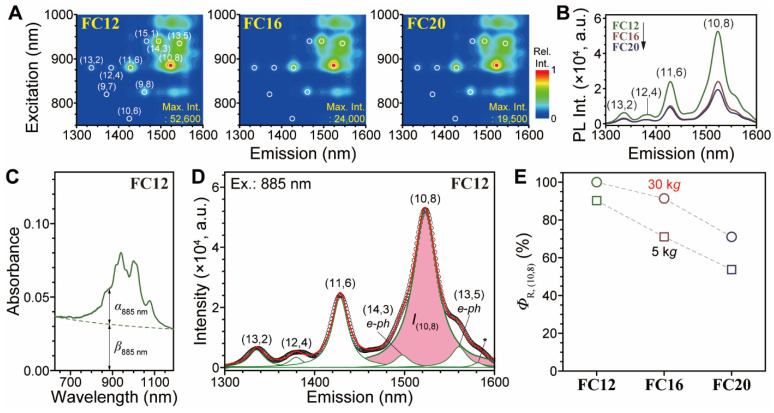
Characterization of *Φ*_R_. (**A**) PLE maps of **FC12**-, **FC16**-, and **FC20**-PSWNT dispersions obtained from 5-k*g* centrifugation. Circle indicates PL positions of SWNT chiralities. (**B**) Overlaid PL emission spectra of PSWNT according to the *l*_S_. Excitation: 885 nm. (**C**) Absorption and (**D**) PL emission spectra obtained from **FC12**-PSWNT dispersion. PL emission spectrum was deconvoluted by Lorentzian shapes and pink shade denotes PL of (10,8) tube. Electron–phonon (*e-ph*) interaction between different SWNT species was denoted. The asterisk symbol indicates a band whose chirality is unknown in our PLE map but is used for increasing the deconvolution accuracy. (**E**) *Φ*_R_, (10,8) according to *l*_S_ and centrifugal force.

Photoluminescence (PL) measurements of PSWNT with increasing flavin *l*_S_ result in lower relative quantum yield (*Φ*_R_) of SWNT owing to increasing CIs. Figure 4A (Figure S10A) shows the PLE maps of **FC8**-, **FC12**-, **FC16**-, and **FC20**-PSWNT dispersions centrifuged at 5 k*g* (30 k*g*). The PLE maps of **FC12**, **FC16**, and **FC20** dispersions show well-resolved sharp PL peaks that are assigned to various SWNT chiralities as indicated [24]. Furthermore, as evidenced by PL emission spectra obtained by excitation at 885 nm, all PLE maps exhibit similar chirality distributions, albeit with lower overall intensity for longer *l*_S_ (Figure 4B and Figure S10B). Among them, (10,8) chirality exhibits the highest PL intensity (*I*_PL_) within the detection limit. Along with the increased CIs in absorption, this result indicates that increased CIs reduces SWNT *I*_PL_. Assuming that (10,8) chirality explains the absorption at 885 nm and that the chirality distribution is the same, we can obtain the relative quantum yield of (10,8), i.e., *Φ*_R,(10,8)_, whose equations are described in Supplementary Materials according to literature [28,33,52]. The (10,8) tube shows the *e*^S^_11_ and *e*^S^_22_ at 1520 nm and 885 nm, respectively. Briefly, *Φ*_R,(10,8)_ is proportional to the normalized area-based *I*_PL_ of (10,8) (*I*_(10,8),normalized_) over absorbance of (10,8) (*α*_(10,8)_). Especially, the background absorption *β*_885nm_ from bundled SWNT and CIs does not contribute to SWNT PL. Therefore, *I*_(10,8), normalized_ instead of *I*_(10,8)_ was utilized to compensate nonradiative contribution given by *I*_(10,8)_/[*α*_885nm_/(*α*_885nm_ + *β*_885nm_)]. The dispersion was diluted such that *e*^S^_22_ of (10,8) dropped below 0.1 absorbance, as shown in Figure 4C [28,52,53]. Figure 4D shows the corresponding Lorentzian deconvoluted PL emission spectrum, and the other samples (Figure S11A–E) were treated similarly. This analysis yielded the values of *α*_885nm_, *β*_885nm_ and *I*_(10,8),normalized_. Figure 4E depicts trends that *Φ*_R,(10,8)_ decreases as the *l*_S_ and centrifugal forces increase. Because CIs are located near SWNT, the PL of SWNT is reduced due to the quenching.

**Figure 5:**
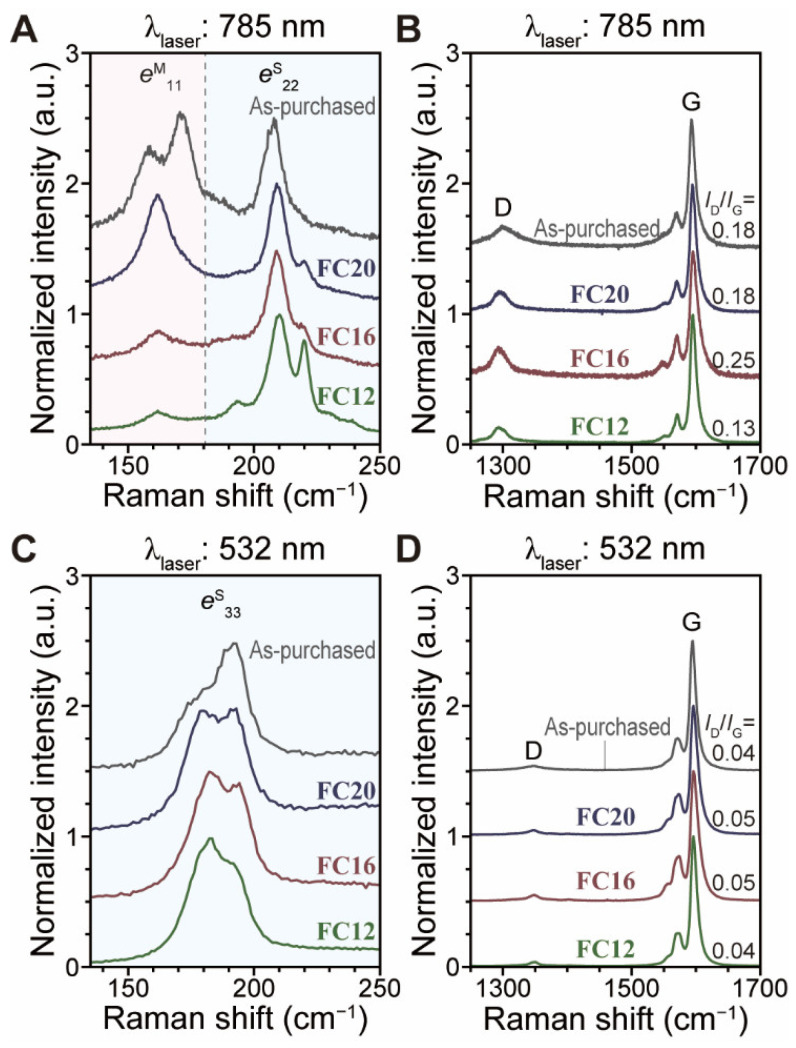
Raman spectrum changes of PSWNT with centrifugation of 5 k*g* with varying *l*_S_ of flavin. (**A**) RBM and (**B**) G band spectra excited by a 785-nm laser. As-purchased PSWNT was used as a control. The dispersions were deposited on a 285-nm-thick SiO_2_/Si substrate and were washed with copious amount of acetone to remove flavins. The corresponding (**C**) RBM and (**D**) G band spectra of the sample excited by a 532-nm laser.

Raman spectroscopy was utilized to confirm the *d*_t_-selectivity and increased *m*-PSWNT resulting from an increasing *l*_S_. The radial breathing mode (RBM) is an out-of-plane tangential vibration mode that is inversely proportional to SWNT *d*_t_ [33,48]. The selectivity was verified by using 785-nm and 532-nm lasers to probe samples of 5 k*g* (Figure 5A–D) and 30 k*g* (Figure S12A–D) dispersed by 0.61 mM in which the 785-nm laser is used to probe smaller-*d*_t_ *s*-SWNT and larger-*d*_t_ *m*-SWNT whereas the 532-nm one is used to probe *s*-SWNT with a larger *d*_t_ range. First, the 785-nm-excited RBM spectra of PSWNT derived from various flavin derivatives (Figure 5A) show that as *l*_S_ increases, PSWNT dispersions show increased intensity of the 165 cm^−1^ band and decreased intensity of the 225 cm^−1^ band originating from respective *e*^M^_11_ and *e*^S^_22_, indicating decreasing smaller-*d*_t_ *s*-SWNT and increasing *m*-SWNT, respectively. This is consistent with the absorption observations. Additionally, the *m*-SWNT bands from **FC12**–**FC20** are smaller than those of as-purchased PSWNT. The resulting D and G bands (Figure 5B) originating from disordered and graphitic vibrations, respectively, are positioned at 1293 and 1595 cm^−1^, respectively [54]. The intensity ratio of D to G bands (*I*_D_/*I*_G_) is the lowest for **FC12** (i.e., 0.13) and highest for **FC16** (i.e., 0.25). The reason for overall higher *I*_D_/*I*_G_ is mainly due to the lower quantum efficiency of the CCD detector in the near IR. When the 532-nm laser is used, RBM regions of PSWNT dispersed by flavins (Figure 5C) show similar RBM bands originating from *e*^S^_33_ regardless of *l*_S_, whereas as-purchased PSWNT show two larger bands at approximately 180–190 cm^−1^. The resulting D and G bands (Figure 5D) are positioned at 1350 and 1596 cm^−1^. While G band positions remain similar regardless of excitation energy, D band positions excited by the 532-nm laser were upshifted compared to those excited by the 785-nm laser due to the energy-dispersive two-phonon process of the D band [54]. Concerning *l*_S,_ samples of 30 k*g* display more systematic trends. Figure S12A shows a 785-nm-excited RBM spectrum region with progressively increasing *m*-SWNT bands at ~165 cm^−1^. Figure S12B displays the D and G bands, wherein *I*_D_/*I*_G_ increases (i.e., 0.20, 0.25, and 0.32) with increasing chain length. As *l*_S_ increases for the 532-nm laser excitation, RBM spectra (Figure S12C) show a systematic increase in the intensity of the 190 cm^−1^ band, and *I*_D_/*I*_G_ value (Figure S12D) increases progressively (i.e., 0.04, 0.05, and 0.06). The slight discrepancy in the trends between samples of 5kg and 30 k*g* appears to stem from sample heterogeneity. Overall, changing **FC12** to **FC20** results in increased *m*-SWNT and *I*_D_/*I*_G_ selectivity, along with larger *d*_t_ *s*-SWNT selectivity. Considering the trace amount of *m*-SWNT, the slight increase in *I*_D_/*I*_G_ with increasing *l*_S_ is attributed to the contribution of increased CIs [48], as observed in absorption spectra.

**Figure 6:**
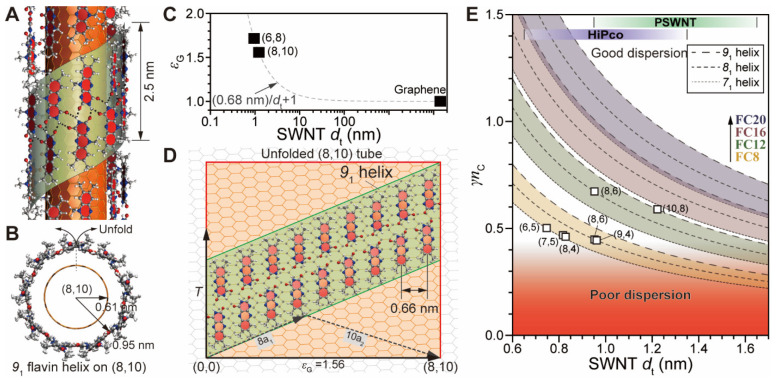
Calculation of the *l*_S_ per carbon in the SWNT using the geometry of a *9*_1_ flavin helix-wrapped (8,10) tube. (**A**) Side view of helical wrapping of flavin spaced with vdW distance (0.34 nm) on (8,10) tube with *d*_t_ = 1.22 nm. SWNT. Note that unit length of flavin helix is 2.5 nm. (**B**) Top view of flavin wrapping on (8,10) tube. (**C**) Plot of SWNT *d*_t_ vs. graphene stretch ratio *ε*_G_ along *C*_h_. Note that each SWNT chirality have different *ε*_G_. (**D**) Projected view of *9*_1_ isoalloxazine helix on graphene sheet with *ε*_G_ = 1.56 while the length of translational vector (*T*) of (8,10) tube remains unchanged. (**E**) Plot of SWNT *d*_t_ vs. *γn*_C_ according to flavin derivatives and helical motifs (*7*_1_, *8*_1_, and *9*_1_ helices). *d*_t_ ranges of HiPco and PSWNT are indicated. Chirality-dependent helical motifs are obtained from Ref. [29].

Considering the *d*_t_ selectivity of flavin derivatives, it would be important to establish *l*_S_ per carbon atom in SWNT based on *d*_t_ to provide stable dispersion (see Materials and Methods section and Supplementary Materials for the detailed geometric modeling). The structural motif adopted in a helical flavin assembly on SWNT is well-known [25,27,28,29,30,31] and has an *8*_1_ flavin helix on (6,8) SWNT [25,27,30,55], whose side and top views are shown in Figure S13A,B. Moreover, the helical motifs for SWNTs with different *d*_t_ values vary, such as *7*_1_, *8*_1_, and *9*_1_ with increasing *d*_t_ [31]. For instance, (6,5) and (8,6) tubes with *d*_t_ values of 0.75 and 0.95 nm, respectively, accommodate *7*_1_ and *8*_1_ flavin helices, suggesting that increasing *d*_t_ by 0.2 nm results in two more flavins in the unit cell. Therefore, (8,10), having *d*_t_ = 1.22 nm and containing 488 carbon atoms in the translational (*T*) length of 3.33 nm, can accommodate *9*_1_ flavin helix with 18 flavins [56,57]. The *9*_1_ helical motif was generated by rotating the flavin dimer by 40°, followed by 2.78-Å translation along the *z* axis by nine times for a unit cell length of 2.5 nm, as shown in the side and top views of Figure 6A,B. The unfolding of concentric SWNT and flavin helix cylinders in Figure 6B causes the inner graphene cylinder to stretch along the chiral vector (*C*_h_) [29]. Figure 6C depicts the graphene stretch ratio (*ε*_G_) according to *d*_t_: *ε*_G_ = (0.68 nm)/(*d*_t_ + 1), which was derived from the ratio between SWNT *d*_t_ and flavin helix diameter, with 0.68 nm denoting twice the vdW distance between the concentric cylinders. The trend indicates that smaller-*d*_t_ SWNT requires higher *ε*_G_. Figure 6D (Figure S13C) depicts a pictorial illustration of an unfolded flavin/(8,10) (and (6,8)) graphene surface with *9*_1_ (*8*_1_) helical flavin arrangement on a two-dimensional graphene sheet stretched by *ε*_G_ = 1.56 [29].

The aforementioned *d*_t_ selectivity based on flavin surfactants creates a relationship for SWNT *d*_t_ vs. *l*_S_ of flavin to promote good SWNT dispersion. A consideration of the *9*_1_ flavin assembly on the stretched graphene surface suggests that the ratio between flavin per carbon atom in a given SWNT (*γ*) exists. For instance, (8,10) has the *γ* value of 0.049 (i.e., ~20 carbon atoms of SWNT corresponding to the footprint of isoalloxazine). The result of multiplying *γ* with the number of carbon atoms in the alkyl side chain of flavin (*n*_C_) is 0.049 × *n*_C_. **FC12** possesses the shortest *n*_C_ required to form stable PSWNT dispersion, with *γn*_C_ with 0.59, suggesting that the carbon atom in SWNT requires 0.59 methylene and methyl groups in the side chain for PSWNT buoyancy. For smaller-*d*_t_ SWNT, the (8,6) tube with *8*_1_ **FC12** helix shows an increased *γn*_C_ value of 0.672 due to the increased *γ* = 0.056 (see Supplementary Materials). Figure 6E depicts *γn*_C_ values for flavin surfactants based on three helical motifs (i.e., *7*_1_, *8*_1_, and *9*_1_ helices). Several SWNT chiralities [i.e., mainly (8,4) and (7,5)] observed with HiPco SWNT dispersion suggest that **FC8** could exhibit a *7*_1_ helix. Therefore, Figure 6E considers the flavin helix polymorphism. The margin and regimes for good SWNT dispersibility are indicated by the red shaded regime and the aforementioned polymorphism. Thus, it can be deduced that the aforementioned larger-*d*_t_ selectivity for flavin with longer *l*_S_ is related to *γn*_C_. For example, the marginal region for poor/good dispersions is more overlapped with larger *d*_t_ SWNT derived from *γn*_C_ of **FC12** than those derived from **FC16** and **FC20**. Therefore, **FC12** is more likely to select SWNT with smaller *d*_t_. In addition, chirality-specific SWNT dispersion observed in the **FC8** case originates from *γn*_C_ situated in the marginal regime between good and poor dispersions. This result suggests that the surfactant with marginal *γn*_C_ is expected to have chiral selectivity.

Next, the reason behind the increase in background absorptions with increasing *l*_S_ of flavin is investigated. The morphology of as-purchased PSWNT was examined by TEM. Figure S14A exhibits the as-purchased PSWNT which contains SWNT, CIs, and metal catalyst. A high magnification TEM image (Figure S14B) reveals that CI is a few-layered defective graphene structure having an interlayer distance of 0.38 nm, as evident by the fast Fourier transform (FFT) image of the inset. SEM measurements revealed CI trends in the case of PSWNT dispersion assisted by flavins (Figure S14C–E). For this, PSWNT films were created by filtration of PSWNT dispersion followed by washing with copious amounts of acetone to remove flavin derivatives. The size and number in CIs, which are highlighted in red, in SWNT films derived from surfactants initially increase from **FC12** to **FC16**, and remain similar from **FC16** to **FC20.** This observation can be attributed to the increased vdW interaction between the alkyl side chain of flavin and flat graphene sidewall of CIs. The longer the alkyl side chain, the more vdW interaction occurs, as shown in Figure 1A. These results are consistent with the absorption results.

**Figure 7:**
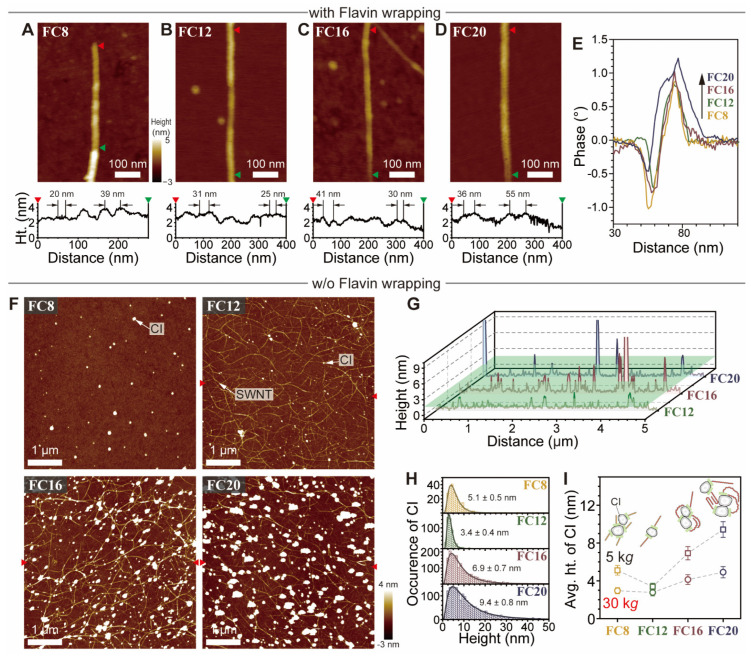
Morphologies of SWNT and CIs in **FC8**-, **FC12**-, **FC16**- and **FC20**-PSWNT dispersions centrifuged at 5 k*g* with or without flavin wrappings. Cases with flavin wrapping: AFM height images of (**A**) **FC8**-, (**B**) **FC12**-, (**C**) **FC16**-, and (**D**) **FC20**-wrapped SWNT on mica. (Bottom) height traces of flavin wrapping on SWNT along the length indicated by triangles in images. (**E**) Their phase traces along the dashed lines as shown in Figure S15A to S15D. Cases without flavin wrapping: (**F**) height images of each dispersion deposited on a 285-nm-thick SiO_2_/Si substrate and (**G**) height profiles extracted from pair of triangles in (**F**). Limit of individualized PSWNT is indicated by a 1.6-nm green plane. (**H**) Height histograms of CIs fitted by using a Lognormal function. Average height and standard deviation are listed. (**I**) Plot of average height of CIs vs. *l*_S_ of the flavin surfactants according to centrifugal force. Cartoons illustrate the interaction of CIs and flavin derivatives.

AFM was used to confirm the effect of flavin *l*_S_ on the morphology of PSWNT and CIs in dispersions. Individual PSWNT wrapped by **FC8**, **FC12**, **FC16**, and **FC20** are confirmed using AFM after gentle *p*-xylene washing to remove extra flavins and subsequent annealing on mica, as shown in Figure 7A–D. The height images show helical grooves formed by flavin wrapping along SWNT with a period of 20–55 nm on top of an average diameter of 2.0 nm of the flavin-PSWNT construct (see line profiles). Moreover, their phase images (Figure S15A–D) show that flavin wrapping with longer *l*_S_ causes greater phase shifts (Figure 7E), which originate from the softness of the longer side chain [58,59]. When the samples are washed with acetone, the flavin-removed [25] topographies are witnessed. Figure 7F depicts the SWNT and CI topographies based on flavin *l*_S_ generated using centrifugation of 5 k*g* and deposited on a 285-nm-thick SiO_2_/Si substrate. While the **FC8**-PSWNT sample contains only CIs, the **FC12**, **FC16**, and **FC20** samples formed from dispersions prepared with centrifugation of 5 k*g* contain random networked SWNT with uniform heights interfaced with CI agglomerates. Interestingly, most CI agglomerates are adsorbed on the SWNT sidewall rather than existing separately on the SiO_2_/Si substrate, which is consistent with previous TEM results [48]. The SWNT morphologies does not significantly change in height, whereas the size and number of CI agglomerates increase with increasing alkyl *l*_S_. These results indicate that an increased *l*_S_ induces larger CI agglomerates but has no effect on SWNT morphologies. A comparison of the height profiles derived from **FC12**–**FC20** (Figure 7G) revealed that increasing *l*_S_ induces more CIs with heights greater than 1.6 nm, which is an upper limit of individualized PSWNT having a *d*_t_ value of approximately 1.3 ± 0.35 nm [33]. These results indicate that the majority of PSWNTs dispersed by *p*-xylene with flavins are dispersed in individual states with CI aggregates on top and that background absorption changes previously discussed originate from variations in the CI content.

Figure 7H shows the height histograms of CIs in flavin-derived SWNT dispersions. CI agglomerates were subjected to size analysis, and the histogram fitting is best described as a lognormal distribution function herein. The dispersions formed using **FC12** exhibit the smallest average height (i.e., 3.4 nm) of CIs and the narrowest height distribution among the four. This finding closely resembles the results of the absorption experiments. Using a similar method, 30 k*g* of prepared samples (Figure S16A–C) were found to exhibit smaller height trends but lesser distributions. Figure 7I shows a plot of the average height of the CIs vs. flavin derivative as a function of the two centrifugal forces. Samples prepared with centrifugation of 5k*g* and 30 k*g* show an initial decrease in CI size, followed by an increase as the length of the alkyl side chain increases. Because CI is an agglomerate form of defective few-layered graphene [48], its size should be enhanced by longer flavin side chains as a consequence of vdW interactions, as shown in Figure 1A. For **FC8**, the limited dispersion of PSWNT promotes higher concentrations of **FC8** in *p*-xylene, resulting in increased adsorption to the CIs and formation of large aggregates. Along with the absorption measurement, the AFM analysis provides a qualitative correlation between the CI content and flavin *l*_S_ due to vdW interaction, indicating that **FC12** is the best for maximizing net SWNT purity.

Moreover, the existing *δ* relationship between the flavin *l*_S_ and CI content contributed to the overall SWNT purity. Solubility parameters *δ* have provided insight into bare SWNT dispersibility in solvents [60,61], SWNT micellarized by various aqueous surfactants [62], and a surfactant-SWNT in various solvents [33]. According to the literature [33,63], minimizing the enthalpy of mixing (*H*_mix_) caused by the difference between *δ* (Δ*δ*) of the solvent and the side chain results in an overall negative Gibbs energy gain, ultimately leading to high purity dispersion of SWNT, which is governed by $H_{mix}=\phi_{1}\phi_{2}{\left(\delta_{1}-\delta_{2}\right)}^{1/2}$, where *φ*_n_ and *δ*_n_ are the mole fractions and *δ* of each component in a mixture, respectively. The Δ values were calculated from each flavin component using the van Krevelen formulation [63] shown in Equation (1),

(1) $$\delta =\sqrt{\delta_{D}^{2}+\delta_{P}^{2}+\delta_{H}^{2}}$$

where *δ*_D_, *δ*_P_, and *δ*_H_ are the molecular attractions due to molar dispersion forces, molar polarization forces, and H-bonding, respectively. Each *δ* subcomponent is derived from the molar volume and energy (or force) according to Equation (2),

(2) $$\delta_{D}=\frac{\Sigma F_{D}}{V_{i}},\;\delta_{P}=\frac{\sqrt{\Sigma F_{P}^{2}}}{V_{i}},\;\delta_{H}=\sqrt{\frac{\Sigma E_{H}}{V_{i}}}$$

where *F*_D_ is the molar attraction constant due to molar dispersion forces, *F*_P_^2^ is the molar attraction constant due to molar polarization forces, *E*_H_ is the H-bonding energy, and *V*_i_ is the group contribution to molar volume.

**Figure 8:**
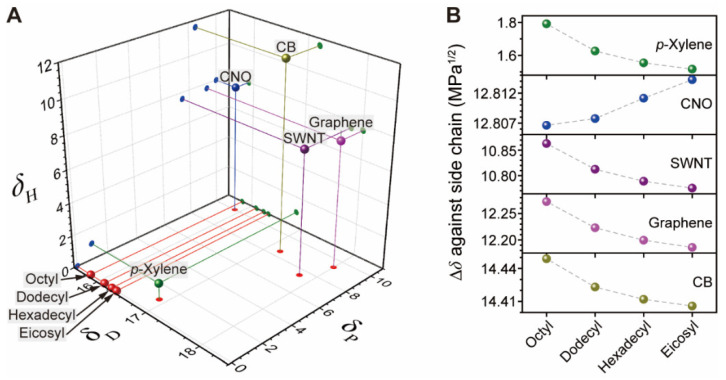
Comparison of *δ* and its subcomponents for flavin side chains, *p*-xylene and representative nano carbon allotropes. (**A**) Comparison of each entity in terms of *δ*_D_, *δ*_P_, and *δ*_H_. (**B**) Δ*δ* of *p*-xylene and nano carbon allotropes against *l*_S_ of flavin.

Table S1 listed *δ* and its subcomponents (*δ*_D_, *δ*_P_, and *δ*_H_) associated with side chains, *p*-xylene [63], and various nanoscale carbon allotropes, including carbon nano-onion (CNO) [64], SWNT [61], graphene [65], and carbon black (CB) [66]. As shown in Figure 8A, with increasing *l*_S_, the *δ* values in three dimension approach those of *p*-xylene along with changes in the subcomponent *δ*_D_. Generally, Δ*δ* < 5 MPa^1/2^ provides good miscibility between components and solvent [63], and Δ*δ* < 1.6 MPa^1/2^ observed for all side chains against *p*-xylene (Table S1) fall within this range. It is noteworthy that graphene, SWNT, and CB have higher *δ*_D_ values than *p*-xylene. Considering the Δ*δ* between various nanocarbons, increasing flavin *l*_S_ results in a reduced Δ*δ* for graphene, SWNT, and CB (Figure 8B). Because CI has a similar crystalline structure to CB, albeit with a few-layered graphene and a much higher *sp*^2^ content [48], the solvent polarity parameter based formulation explains why flavins with longer *l*_S_ promote the formation of greater amounts of CIs. Overall, a comparison of Δ*δ* is in accord with the observed dispersion trends for SWNT and CIs.

**Figure 9:**
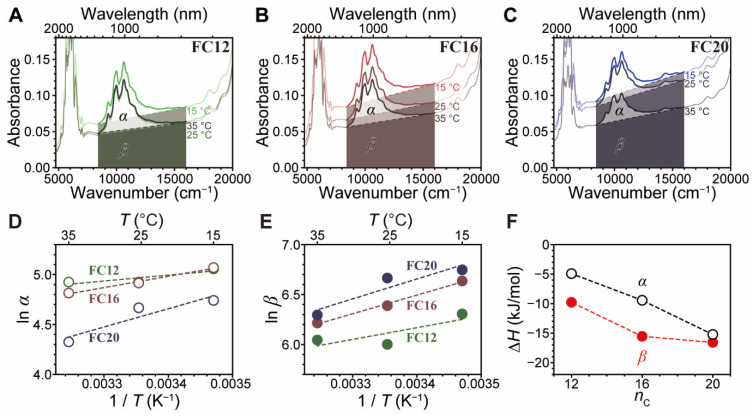
Sonication bath temperature dependent absorption spectrum change according to *l*_S_. Changes of *α* from (**A**) **FC12**-, (**B**) **FC16**-, and (**C**) **FC20**-PSWNT dispersions which were prepared by sonication at 15, 25, and 35 °C (from light to dark colors), respectively. Gray shades denote *β*. van’t Hoff plots for (**D**) *α* and (**E**) *β* from **FC12**- (green), **FC16**- (purple), and **FC20**-derived dispersions (blue) at 940 nm vs. reciprocal sonication temperatures. The solid lines show the linear regressions from each point. (**F**) Δ*H* vs. *α* and *β* according to number of carbon atom in flavin side chain (*n*_c_). The dotted lines are drawn for visual guidance.

Control of sonication bath temperature, which has been utilized to control SWNT dispersibility [35,37], enables further reduction of the co-dispersed CI concentration. Figure 9A–C show the absorption spectra of SWNT dispersion formed using **FC12**, **FC16**, and **FC20** and sonication at three temperatures of 15, 25, and 35 °C. For **FC12**-PSWNT dispersion, with increasing sonication temperature, *β* is greatly reduced compared to *α*, which is maintained almost constant. Conversely, **FC16** and **FC20** reduce both *α* and *β* with increasing temperatures. To understand this behavior in terms of temperature and *l*_S_, we considered the following ideal chemical equilibrium *ν*flavin(*sol*) + SWNT(*s*) ⇌ *ν*flavin-SWNT(*sol*), where *ν* is number of flavins associated with an SWNT. The equilibrium constant for this process is *K* = [*ν*flavin-SWNT]/[flavin]*^ν^*. Since *ν*flavin-SWNT is proportional to absorbance *α*, the enthalpy change (Δ*H*) of the reaction can be obtained using the van’t Hoff equation:

(3) $$\ln\;K=-\frac{\Delta H}{RT}+\Delta S/R$$

where Δ*S* is the entropy change, *R* is the gas constant (8.314 J/mol·K), and *T* is the absolute temperature. *β* can be treated in a similar manner. Here, *K* is proportional to the concentration of *ν*flavin-SWNT.

The plots of the temperature-dependent changes in *α* and *β* (Figure 9D,E) show that increasing the sonication bath temperature greatly reduces *β* compared to *α*, irrespective of the flavin *l*_S_. This is partially consistent with the temperature dependency of polythiophene-SWNT dispersion [37]. The slopes of these plots, which correspond to −Δ*H*/*R*, are all positive. Using this data, Δ*H* trends for *α* and *β* according to *l*_S_ (Figure 9F) were obtained. The data show that Δ*H* associated with *α* linearly decreases from −5 to −15 kJ/mol with increasing *l*_S_, whereas Δ*H* associated with *β* has a minimum at **FC16** and **FC20**. This result indicates that SWNT along with CIs are stabilized by a flavin possessing a longer side chain. Similarly, the Δ*H*s associated with *α* and *β* of the **FC16**- and **FC20**-derived PSWNT dispersions originate from different side chain configurations (i.e., hairpin-folded or interdigitated structures) because flavin-SWNT have radially extended side chains in solvents, such as *p*-xylene [28,33], whereas flavin on disk-like CIs exhibit vertically extended side chain and promotes interaction with adjacent flavin-functionalized CIs, as depicted in cartoon in the AFM results (Figure 7I). The enthalpic change of CIs with increasing *l*_S_ stems from possible interdigitation of the vertically extended *n*-alkyl flavin side chains between CIs at the expense of solvation.

**Figure 10:**
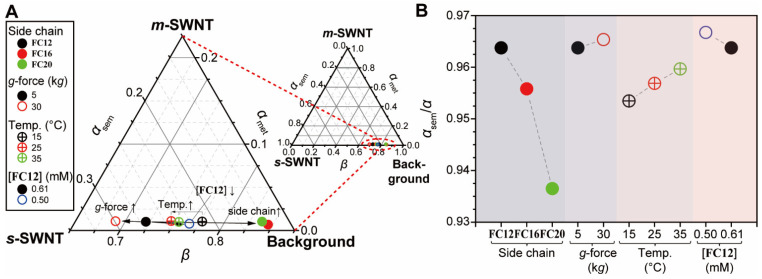
Trends of various parameters (*l*_S_, *g*-force, temperature, and **FC12** concentration) affecting *α*_sem_, *α*_met_, and *β*. (**A**) Ternary plot of *α*_sem_, *α*_met_, and *β* based on 0.61 mM **FC12**-PSWNT dispersion at room temperature. Inset: its full scale. Scales are read in a counter-clockwise manner. (**B**) *α*_sem_/(*α*_sem_ + *α*_met_) plot according to various parameters.

The aforementioned dispersions prepared under various conditions were compared. Each dispersion was analyzed in terms of *α*_sem_, *α*_met_, and *β*. Using 0.61 mM **FC12**-PSWNT dispersion at room temperature as reference, Figure 10A shows a ternary plot of *α*_sem_, *α*_met_, and *β* located at each vertex. Most contributions originate from *β*, which ranges from 69% to 84%, irrespective of conditions. Within our experimental scope, decreasing *l*_S_, increasing sonication bath temperature, increasing *g*-force, and decreasing **FC12** concentration result in decreased *β*. Among them, varying side chain results in a dramatic change in *β*. Moreover, Figure 10B depicts the purity of *s*-SWNT which ranges from 93.5% to 96.5%. Particularly, a lower **FC12** concentration and higher *g*-force lead to an increased *α*_sem_/*α* owing to the *s*-SWNT preference, whereas increasing *l*_S_ and decreased sonication temperature show the opposite trend. The self-assembly nature of flavin explains the behaviors caused by the extrinsic parameter. The concerted H-bonding and π–π interaction between flavins and SWNT [27] are known to be much stronger than vdW interaction between flavin derivatives and defective CI. Therefore, thermal energy induces a larger disruption of vdW-interacted CIs.

**Figure 11:**
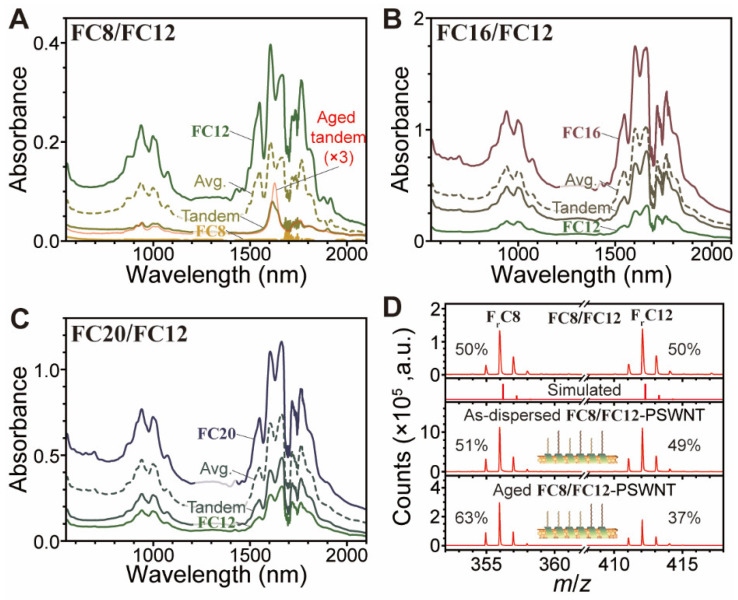
Absorption spectra of PSWNT dispersions generated using isomolar tandem mixtures of flavin surfactants (**A**) **FC8/FC12**, (**B**) **FC16/FC12**, and (**C**) **FC20/FC12**, compared to those from respective single surfactant dispersion, averaged, and aged tandem samples. All measurements except the aged dispersion were acquired by using a cuvette of beam path 10 mm. The average spectra were obtained by averaging each single flavin spectrum. (**D**) MALDI-TOF MS spectra of (top) isomolar **FC8**/**FC12** solution, (middle) surfactants present on the as-prepared **FC8**/**FC12**-PSWNT, and (bottom) surfactants present on the aged **FC8**/**FC12**-PSWNT.

Thus far, the effects of a single flavin surfactant on *d*_t_-dependent SWNT dispersion have been investigated. However, we discovered that a tandem mixture of two flavins can further fine-tune the *d*_t_-distribution of SWNT by utilizing self-assembly of tandem flavins. Figure 11A–C show the absorption spectra of PSWNT dispersions prepared by using combinations **FC8**/**FC12**, **FC16**/**FC12**, and **FC20**/**FC12** containing isomolar amounts of two flavins, including those from the individual and averaged and aged samples. In tandem combinations, all dispersions have much lower absorbances than the average spectra. The fact that SWNT contents generated by tandem flavins have a propensity towards flavin with shorter *l*_S_ demonstrates the significance of flavin solubility. Another aspect to consider is the lower *d*_t_ propensity of the dispersed SWNT derived from the **FC8**/**FC12** tandem mixture. Evidently, the *e*^S^_11_ band at 1625 nm is larger than that of **FC12**-derived dispersion and it was further strengthened in the aged sample (i.e., three years old). The corresponding PLE maps (Figure S17A–C) and PL emission spectra (Figure S17D) clearly indicate the *d*_t_ distribution change of PSWNT when tandem surfactants are combined. In addition, at both centrifugal forces, such aging effects were not significant for single surfactant-SWNT dispersions (Figure S18A,B). Moreover, the aforementioned aged sample with different chirality selection implies that dynamic equilibrium is involved. This is consistent with the observation reported by Mollahosseini et al. [67] that a mixture of 99% **FC12** and 1% flavin functionalized with a fullerene derivative (PCBM) promotes heterostructured flavin-SWNT dispersion formation, whereas the one comprised of 100% flavin functionalized with PCBM does not produce a SWNT dispersion.

The ratio of tandem surfactant on SWNT is disproportionated when compared to the 1:1 flavin ratios obtained by MALDI-TOF MS. Herein, three samples were prepared: an isomolar solution of **FC8**/**FC12**, the as-prepared **FC8**/**FC12** on PSWNT, and the aged **FC8**/**FC12** on PSWNT without free flavins (see Materials and Methods section). It is noteworthy that the **FC8**/**FC12** composition on PSWNT was obtained by filtration of the SWNT and subsequent redispersion of SWNT while free flavins were removed. The MS spectrum of the control shows that **FC8**/**FC12** mixture exhibits the strongest peaks at 356 and 412 *m*/*z* with 50:50 ratio, and their molecular weights are two atomic units larger than those of **FC8** and **FC12** (i.e., 354.45 and 410.55 g/mol, respectively). This result suggests that 355-nm pulsed laser irradiation during the MALDI-TOF process induces photoreduction of **FC8** and **FC12** (ref: [24,68]) into the corresponding reduced forms (**F_r_C8** and **F_r_C12**, the chemical structures of which are shown in Figure S19A,B, respectively) as opposed to no change in dithranol as reference (Figure S19C,D). However, the **FC8**/**FC12** ratios on SWNT in the as-prepared and aged samples increase from 51/49 to 63/37, indicating an increased population of **FC8** on SWNT via the dynamic equilibrium process. The question is why **FC8** is being driven to the SWNT surface. We attributed this behavior to the relatively low solubility of **FC8** among flavins. Figure S20A,B show the absorption spectra of entire flavin surfactants in *p*-xylene and the derived solubilities in *p*-xylene using extinction coefficient of flavin (12,600 L/mol·cm) [40,41]. **FC12** shows higher molar solubility (2.32 mM) than others (1.51, 1.99, and 1.93 mM for **FC8**, **FC16** and **FC20**). The lower solubilities for **FC16** and **FC20** appear to originate from the aforementioned ‘hairpin’ folding of *n*-alkyl chain greater than *n*-dodecyl [69,70].

**Figure 12:**
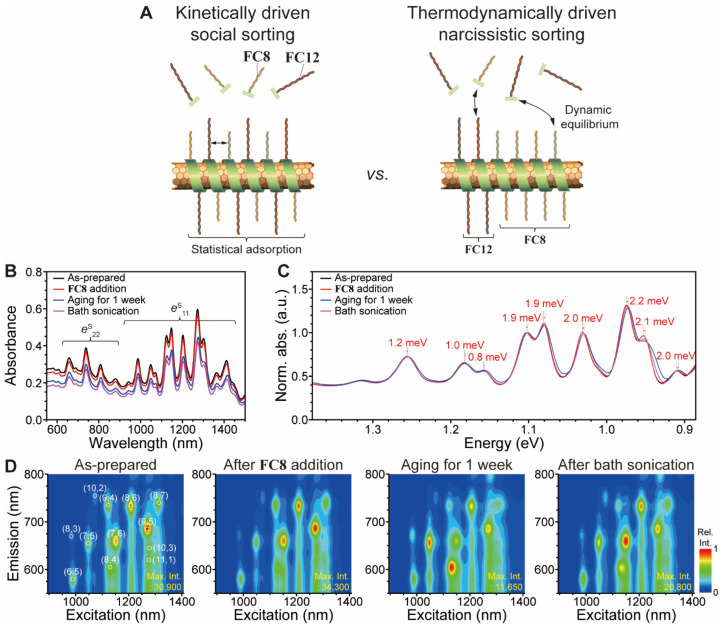
Social or narcissistic sortings of tandem **FC8**/**FC12** on SWNT affecting *d*_t_ enrichment of SWNT dispersions. (**A**) Schematic of social sorting (left) and narcissistic sorting (right) modes of flavin derivatives on SWNT surface. (**B**) Comparison of absorption spectra from the as-prepared **FC12**-PSWNT dispersion (black), and after the addition of isomolar **FC8** (red), after the one-week aged (blue) and after the bath-sonicated sample (magenta). All absorption measurements were acquired by using a cuvette of beam path 10 mm. (**C**) The corresponding normalized *e*^S^_11_ absorption spectra with *d*_t_-dependent redshift degree of the aged sample against 1.1 eV band. (**D**) PLE maps of **FC12**-PSWNT and **FC8**/**FC12**-PSWNT dispersions with addition, aging, and bath sonication treatments. It is noteworthy that enriched chiralities of SWNT are different.

Helical flavin assembly on SWNT can be viewed as templated self-assembly process on SWNT. Self-sorting of surfactants would occur in the case of dual surfactants. Self-sorting behavior from tandem surfactants is either social sorting or narcissistic sorting, as shown in schematic of Figure 12A [71,72,73,74]. Social sorting is a statistical distribution of dissimilar assembly motifs, whereas narcissistic assembly generates homologous assembly through self-recognition. Because of the sonication-assisted self-assembly of flavins, as-prepared **FC8**/**FC12**-PSWNT dispersion is regarded as statistical 1:1 social sorting mainly by a kinetic process, where sonication [45] is an enormous energy source for self-assembly [75]. Meanwhile, during the aging period, **FC8**/**FC12** on PSWNT dispersion undergoes dynamic equilibrium between bound flavins on SWNT and ‘free’ **FC8**/**FC12** in solution, as depicted in Figure 12A, and **FC8** is narcissistically enriched on SWNT.

The aged sample displayed narcissistic sorting. As shown in Figure 12B, 0.61 mM **FC12**-HiPco dispersion was subjected to **FC8** addition, aging, and bath-sonication. The absorption spectrum (black trace) shows well-resolved *e*^S^_11_ and *e*^S^_22_. While addition of **FC8** does not yield an immediate spectral change (red trace) except lowered absorbance, one-week aged samples show a *d*_t_-dependent redshift of *e*^S^_11_ up to 2.2 meV for larger *d*_t_ SWNT possibly due to small SWNT bundling, as shown in the electronvolt scale (Figure 12C). The absorption spectrum (magenta trace) was then restored to its original *e*^S^_11_ positions after a brief bath sonication. The corresponding PLE maps (Figure 12D) show such *d*_t_-dependent changes for such treatments. First, the as-prepared sample exhibits (9,5), (8,6), and (7,5) enrichment as major chiralities and does not change significantly after **FC8** addition. However, the aging period induces (8,4) and (7,5) enrichment by lowering *I*_PL_ of the larger *d*_t_ SWNT (see the max *I*_PL_ of each map). Brief bath-sonication of this sample partially recovered the PL-based SWNT abundance (i.e., (9,5), (8,6), and (7,5) enrichment) similar to that of the as-prepared sample. Considering the aforementioned smaller-*d*_t_ SWNT selectivity by **FC8**, the aging of **FC8**/**FC12**-HiPco sample induces narcissistic sorting of **FC8** on SWNT surface.

**Figure 13:**
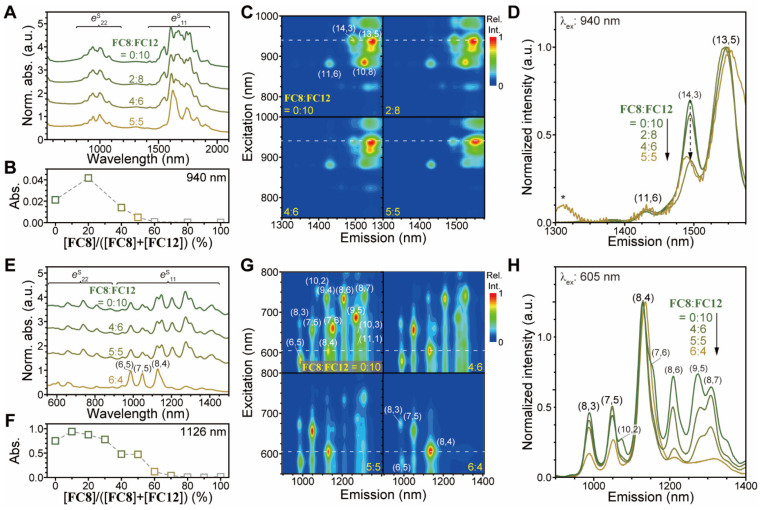
Kinetically driven chirality enrichment behavior of **FC8**/**FC12** with PSWNT and HiPco SWNT types. PSWNT: (**A**) The absorption spectra of PSWNT prepared by varying ratios of **FC8**/**FC20** while the total concentration of flavins remains the same (0.61 mM). The spectra were normalized against maximum *e*^S^_22_ bands at 940 nm and offset by same interval. (**B**) Absorbance trend of the 1623-nm peak according to the ratio. (**C**) The corresponding PLE maps and (**D**) PL emission spectra of PSWNT according to the ratio. HiPco: (**E**) The absorption spectra of HiPco prepared by varying ratios of **FC8**/**FC20** while the total concentration of flavins remains the same (2.44 mM). (**F**) Absorbance trend of the 1126-nm peak according to the ratio. (**G**) The corresponding PLE maps and (**H**) PL emission spectra of PSWNT according to the ratio.

Next, we investigated social sorting by varying the **FC8**/**FC12** ratio for PSWNT and HiPco dispersions. Figure 13A shows the absorption spectrum change by changing **FC8**/**FC12** molar ratio from 0:10 to 5:5 with the concentration maintained at 0.61 mM. Any increase of the **FC8** ratio above 5 does not yield a stable dispersion, setting the lower limit for stable PSWNT dispersion. Evidently, increasing the **FC8** portion over **FC12** promotes *d*_t_ distribution narrowing whose *e*^S^_11_ is centered at approximately 1625 nm. Overall absorbance at 940 nm as reference decreases with an increasing **FC8** ratio, as evident by Figure 13B. The corresponding PLE maps (Figure 13C) show that with an increasing **FC8** ratio, *I*_PL_ of (13,5) chirality at 1625 nm increases, whereas that of (10,8) chirality decreases. The corresponding normalized PL emission spectra (Figure 13D) exhibit (13,5) enrichment. The usage of HiPco having smaller average *d*_t_ results in few chirality enrichments of SWNT in both absorption and PL spectroscopies (see Figure S21A to S21M for photograph of the dispersion, entire absorption, and PLE maps). Figure 13E shows the absorption spectrum change of HiPco dispersion with an increasing **FC8** ratio. When **FC8**:**FC12** is equal to 6:4, few chirality enrichments such as (8,4), (7,6), (6,5), and (8,3) occur albeit the reduced absorbance (Figure 13F). Notably, this absorbance exhibits a very low background. These results indicated that the marginal dispersibility of SWNT induced by surfactants is a key parameter for both selection of SWNT chiralities and exclusion of CIs. The corresponding PLE maps (Figure 13G) pinpoint the SWNT chiralities enriched during this process. While the initial PLE map from **FC8**/**FC12** = 0/10 displays the denoted 12 different SWNT chiralities, the 6:4 sample mainly shows the four SWNT chiralities. PL emission (Figure 13H) further supports such few chirality enrichments.

## 4. Conclusions

In this study, we investigated the single/tandem effects of flavin *l*_S_ on the purity, *m*-/*s*-ratio, and *d*_t_ selectivity of SWNT using isomolar flavin concentration. A two-step sequence was used to synthesize **FC8**, **FC12**, **FC16**, and **FC20**. In *p*-xylene, **FC12**, **FC16**, and **FC20** produced stable *s*-enriched PSWNT dispersions, whereas **FC8** produced a poor one owing to shorter *l*_S_. **FC8** dispersion with a smaller average *d*_t_ SWNT revealed specific chirality selection towards smaller *d*_t_. Flavins with longer *l*_S_ exhibited s-SWNT dispersion with higher CIs and *m*-SWNT. Considering helical flavin wrapping motifs on the SWNT, we empirically derived ‘*l*_S_ per carbon atom in SWNT’ to provide stable SWNT dispersion according to SWNT *d*_t_. Moreover, morphology change of CIs according to increasing *l*_S_ was ascribed to the vdW interaction and side-chain configurations. The smaller solubility parameter difference was ascribed to the apparent increase in CIs, which affected the quantum yield of SWNT due to fluorescence quenching of nearby metallic CIs. Increasing sonication bath temperatures enables flavin derivatives to disperse CIs less efficiently than SWNTs. Based on this, the enthalpy changes of both SWNT and CIs were derived according to *l*_S_. The overall purity of SWNT over CIs increased with decreasing *l*_S_ and **FC12** concentration, increasing *g*-force and sonication bath temperature. A tandem mixture of the flavin surfactants was employed to fine-tune SWNT dispersibility and *d*_t_-selectivity via either social or narcissistic sorting. While the as-prepared tandem surfactants exhibit social sorting behavior with statistical adsorption, the aged tandem surfactants on SWNT exhibited narcissistic sorting of flavin with shorter *l*_S_ owing to the lower solubility. Increasing the proportion of flavin with lower solubility induces SWNT *d*_t_ narrowing towards smaller *d*_t_ and promotes few chirality enrichments upon using smaller average *d*_t_ SWNT. These findings address an important aspect of unanswered *l*_S_ in surfactant design for *s*-SWNT purity control. The *d*_t_ modulation concepts uncovered in this study should be deployable for optoelectronic and energy applications, such as thin-film transistor and thermoelectric applications, dependent upon *d*_t_.

## Data Availability

The data presented in this study are available on request from the corresponding author.

## Supplementary Materials

The following supporting information can be downloaded at: https://www.mdpi.com/article/10.3390/nano12193380/s1, Scheme S1: Sideview (left) and topview (right) of 91 flavin helix-wrapped (8,10) tube (**A**) before and (**B**) after geometry optimization. Lumiflavin (methyl flavin) was utilized for molecular mechanics (MM) simulation.; Figure S1: ^1^H NMR and ^13^C NMR spectra of **1**-**4**; Figure S2: ^1^H NMR and ^13^C NMR spectra of **1**-**4**; Figure S3: ^1^H NMR and ^13^C NMR spectra of **FC8**, **FC12**, **FC16**, and **FC20**; Figure S4: ^1^H NMR and ^13^C NMR spectra of **FC8**, **FC12**, **FC16**, and **FC20**; Figure S5: absorption spectrum and corresponding PLE map of 2.44 mM **FC12**-HiPco; Figure S6: larger *d*_t_ SWNT propensity of flavin with longer *l*_S_; Figure S7: dispersion of PSWNT with low flavin concentration; Figure S8: defining *α* and *β* in the absorption spectra in wavenumber scale; Figure S9: comparison of PSWNT dispersions according to centrifugal forces; Figure S10: PLE maps of **FC12**-, **FC16**-, and **FC20**-PSWNT dispersions centrifuged at 30 k*g*; Figure S11: comparison of absorption and PL emission spectra for *Φ*_R_, _(10,8)_ determinations; Figure S12: Raman spectrum change of PSWNT with 30 k*g* centrifugation; Figure S13: calculation of the *l*_S_ per carbon atom in the SWNT; Figure S14: variation of CI present in PSWNT films prepared by varying flavin *l*_S_; Figure S15: the corresponding AFM phase images of flavin-wrapped PSWNT; Figure S16: SWNT and CI morphology trends in **FC8**-, **FC12**-, **FC16**-, and **FC20**-PSWNT; Figure S17: PLE maps of PSequationWNT dispersions by using tandem flavin surfactants; Figure S18: comparison of absorption spectra from the as-prepared and the aged samples; Figure S19: chemical structures in MS spectra; Figure S20: solubility determination of flavin derivatives by using absorption spectra; Figure S21: effect of **FC8**/**FC12** ratio on *d*_t_ distribution of HiPco dispersion; Entire and compound spectra of FrC8, FrC12, FrC16, and FrC20 acquired by MALDI-TOF MS; Table S1: *δ* and its subcomponents.
