# Supplementary material for: The Effects of Lengths of Flavin Surfactant N-10-Alkyl Side Chains on Promoting Dispersion of a High-Purity and Diameter-Selective Single-Walled Nanotube
Source: Nanomaterials (Basel). 2022 Sep 27;12(19):3380. doi: 10.3390/nano12193380 (PMC9565467; doi:10.3390/nano12193380)
Supplement: Supplementary file 1 [file nanomaterials-12-03380-s001.zip › nanomaterials-1909900-supplementary.pdf]

nanomaterials

MDPI

Supplementary Materials for

The Effects of Lengths of Flavin Surfactant N-10-Alkyl Side Chains on Promoting Dispersion of a High-Purity and Diameter-Selective Single-Walled Nanotube

Minsuk Park, Seongjoo Hwang and Sang-Yong Ju *

Department of Chemistry, Yonsei University, 50 Yonsei-ro, Seodaemun-gu, Seoul 03722, Korea
* Correspondence: syju@yonsei.ac.kr; Tel.: +82-2-2123-5639

Citation: Park, M.; Hwang, S.; Ju, S.-Y. The Effects of Lengths of Flavin Surfactant N-10-Alkyl Side Chains on Promoting the Dispersion of a High-Purity and Diameter-Selective Single-Walled Nanotube. *Nanomaterials* **2022**, *12*, 3380. https://doi.org/10.3390/nano12193380

Academic Editor: Catherine Journet

Received: 26 August 2022
Accepted: 23 September 2022
Published: 30 September 2022

Publisher's Note: MDPI stays neutral with regard to jurisdictional claims in published maps and institutional affiliations.

Copyright: © 2022 by the author. Licensee MDPI, Basel, Switzerland. This article is an open access article distributed under the terms and conditions of the Creative Commons Attribution (CC BY) license (https://creativecommons.org/licenses/by/4.0/).

Synthesis of flavin derivatives:

N[1]-dodecyl-4,5-dimethylbenzene-1,2-diamine (**2**) and **FC12** were synthesized *via* two-step sequences according to the previously reported method [1], which are analogous to the following synthetic descriptions.

N[1]-Octyl-4,5-dimethylbenzene-1,2-diamine (**1**): 1-chlorooctane (1.49 g, 10 mmol) was added *via* syringe to a dissolved mixture of 4,5-dimethyl-1,2-phenylenediamine (4.08 g, 30 mmol) in triethylamine (TEA) (20 mL) stirred at 130 °C with reflux condenser, and the final mixture was stirred at 130 °C for 12 h under nitrogen atmosphere to prevent oxidation. After cooling and adding dichloromethane (MC, 100 mL), the organic solution was washed with aqueous 10 wt.% Na_2CO_3 solution (40 mL) to remove any salt. The aqueous layer was extracted twice with MC (2 × 100 mL) which was combined with the previously extracted MC. The combined organic extracts were dried over anhydrous $MgSO_4$ and rotary evaporated to dryness. The thin layer chromatography (TLC) retention factor (R_f) of compound **1** was 0.54 with 95:5 MC/methanol (MeOH) eluent. Compound **1** was purified by flash column chromatography on silica gel with 95:5 MC/MeOH as mobile phase to produce 0.805 g (3.24 mmol) of reddish crystals (yield: 32%); m.p.: 53 °C; ^{1}H NMR ($CDCl_3$ with TMS, 400 MHz, Figure. S1A) chemical shift (δ): 6.52 (1H, s), 6.46 (1H, s), 3.16 (NH and NH_2, 3H, br s), 3.06 (2H, t, J = 7.14 Hz), 2.17 (3H, s), 2.13 (3H, s), 1.65 (2H, quintet, J = 14.6 Hz), 1.42 (2H, quintet, J = 13.6 Hz), 1.28 (24H, s), and 0.89 (3H, t, J = 6.83 Hz); ^{13}C NMR ($CDCl_3$ with TMS, 100 MHz, Figure. S2A) δ 136.0, 131.8, 128.1, 125.9, 118.4, 113.9, 44.7, 31.9, 29.8, 29.5, 29.3, 27.3, 22.7, 19.3, 18.8, and 14.1; Anal. Calcd. for $C_{16}H_{28}N_2$ (formula weight (F.W.) = 248.41): C, 77.36; H, 11.36; and N, 11.28;. Found: C, 79.06; H, 13.31; and N, 8.00.

N[1]-Hexadecyl-4,5-dimethylbenzene-1,2-diamine (**3**): 1-chlorohexadecane (2.60 g, 10 mmol) was added through syringe to a dissolved mixture of 4,5-dimethyl-1,2-phenylenediamine (4.08 g, 30 mmol) in TEA (20 mL). A similar protocol for **1** was utilized to synthesize **3**. The R_f value of **3** was 0.84 with 95:5 MC/MeOH. Compound **3** was purified by column chromatography on silica gel in 95:5 MC/MeOH to produce 1.652 g (4.58 mmol) of reddish crystals (yield: 46%); m.p.: 53 °C; ^{1}H NMR ($CDCl_3$ with TMS, 400 MHz, Figure. S1C) δ 6.53 (1H, s), 6.47 (1H, s), 3.17 (NH and NH_2, 3H, br s), 3.06 (2H, t, J = 7.12 Hz), 2.18 (3H, s), 2.13 (3H, s), 1.65 (2H, quintet, J = 14.5 Hz), 1.42 (2H, quintet, J = 13.5 Hz) , 1.27 (24H, s), and 0.88 (3H, t, J = 6.64 Hz); ^{13}C NMR ($CDCl_3$ with TMS, 100 MHz, Figure. S2C) δ 136.0, 131.8, 128.1, 125.8, 118.4, 113.9, 44.7, 31.9, 29.8, 29.7, 29.6, 29.5, 29.4, 27.3, 22.7, 19.3, 18.8, and 14.1; Anal. Calcd. for $C_{24}H_{44}N_2$ (F.W. = 360.62): C, 79.93; H, 12.30; and N, 7.77;. Found: C, 79.06; H, 13.31; and N, 8.00.

N[1]*-Eicosyl-4,5-dimethylbenzene-1,2-diamine* (**4**): 1-chloroeicosane (3.17 g, 10 mmol) was added through syringe to a completely dissolved mixture of 4,5-dimethyl-1,2-phenylenediamine (4.08 g, 30 mmol) in 20 mL TEA. A similar protocol used for **1** was utilized to synthesize **4**. The R_f of **4** was 0.86 with 95:5 MC/MeOH. Compound **4** was purified by flash column chromatography on silica gel in 95:5 MC/MeOH to produce 1.040

g (2.50 mmol) of reddish crystals (yield: 25%); m.p.: 54 °C; ^{1}H NMR ($CDCl_3$ with TMS, 400 MHz, Figure. S1D) δ 6.52 (1H, s), 6.47 (1H, s), 3.21 (NH and NH_2, 3H, br s), 3.06 (2H, t, J = 7.2 Hz), 2.17 (3H, s), 2.13 (3H, s), 1.65 (2H, quintet, J = 14.5 Hz), 1.42 (2H, m) , 1.26 (32H, s), and 0.88 (3H, t, J = 6.6 Hz); ^{13}C NMR ($CDCl_3$ with TMS, 100 MHz, Figure. S2D) δ 135.8, 131.8, 128.1, 126.0, 118.4, 113.9, 44.7, 31.9, 29.7, 29.7, 29.7, 29.7, 29.7, 29.7, 29.7, 29.7, 29.7, 29.7, 29.7, 29.7, 29.7, 29.5, 29.4, 27.3, 22.7, 19.3, 18.8, and 14.1; Anal. Calcd. for $C_{28}H_{52}N_2$ (F.W. = 416.73): C, 80.70; H, 12.58; and N, 6.72;. Found: C, 81.88; H, 14.06; and N, 7.29.

10-Octyl-7,8-dimethyl-10H-benzo[g]pteridine-2,4-dione (**FC8**): A similar protocol used for synthesis of **FC12** [1] was utilized except for using compound **1**. Briefly, a mixture of **1** (0.472 g, 1.9 mmol), alloxan monohydrate (0.320 g, 2 mmol), boric oxide (0.42 g, 6 mmol), and glacial acetic acid (60 mL) was stirred at 60 °C for 6 h, showing gradual formation of yellow heterogeneous mixture. The resulting mixture was diluted with deionized water (500 mL) and the yellow precipitate was collected by filtration and vacuum-dried. The R_f of the target compound was 0.22 with 95:5 MC/MeOH. **FC8** was purified by flash column chromatography on silica gel in 95:5 MC/MeOH to produce 0.376 g (1.06 mmol) of yellow crystals (yield: 56%); m.p.: 225 °C; ^{1}H NMR ($CDCl_3$ with TMS, 400 MHz, Figure. S3A) δ 8.39 (1H, s), 8.07 (1H, s), 7.39 (1H, s), 4.69 (2H, br s), 2.57 (3H, s), 2.46 (3H, s), 1.86 (2H, quint, J = 15.6 Hz), 1.53 (2H, quint, J = 15.3 Hz), 1.40 (2H, m), 1.29 (6H, s), and 0.89 (3H, t, J = 7.0 Hz); ^{13}C NMR ($CDCl_3$ with TMS, 100 MHz, Figure. S4A) δ 159.6, 155.2, 150.0, 148.3, 137.1, 136.1, 135.0, 132.8, 131.1, 115.3, 45.4, 31.7, 29.3, 29.3, 27.1, 26.8, 22.6, 21.8, 19.6, and 14.1; Anal. Calcd. for $C_{20}H_{26}N_4O_2$ (F.W. = 354.45): C, 67.77; H, 7.39; and N, 15.81; O, 9.03;. Found: C, 68.57; H, 7.42; and N, 15.95. MALDI-TOF MS: Calcd. for $C_{20}H_{26}N_4O_2$ m/z 354.21 (100 %), 355.21 (22.0 %), 356.21 (3.0%), Obtained as reduced form: 354.94 (30.7 %), 355.94 (100 %), 356.95 (32.9%); FTIR (KBr pellet): ν (cm^{-1}) 3410, 2924, 2855, 1694, 1663, 1580, 1542; Raman (785 nm excitation, shift in cm^{-1}): 288, 421, 498, 605, 679, 740, 1230, 1350.

10-Hexadecyl-7,8-dimethyl-10H-benzo[g]pteridine-2,4-dione (**FC16**): A mixture of **3** (0.685 g, 1.9 mmol), alloxan monohydrate (0.320 g, 2 mmol), and boric oxide (0.42 g, 6 mmol) in 60 mL glacial acetic acid was stirred at 60 °C for 6 h. A similar protocol used for synthesis of **FC8** was utilized. The TLC R_f of the target compound was 0.30 with 95:5 MC/MeOH. **FC16** was purified by column chromatography on silica gel in 95:5 MC/MeOH to produce 0.471 g (1.01 mmol) of yellow crystals (yield: 53%); m.p.: 218 °C; ^{1}H NMR ($CDCl_3$ with TMS, 400 MHz, Figure. S3C) δ 8.37 (1H, s), 8.07 (1H, s), 7.39 (1H, s), 4.69 (2H, br s), 2.57 (3H, s), 2.46 (3H, s), 1.86 (2H, quint, J = 15.9 Hz), 1.52 (2H, quint, J = 15.5 Hz), 1.39 (2H, m), 1.26 (22H, s), and 0.88 (3H, t, J = 7.0 Hz); ^{13}C NMR ($CDCl_3$ with TMS, 100 MHz, Figure. S4C) δ 159.6, 155.3, 150.0, 148.3, 137.1, 136.1, 135.0, 132.8, 131.1, 115.3, 45.4, 31.9, 31.9, 29.7, 29.7, 29.7, 29.7, 29.7, 29.7, 29.7, 29.7, 29.5, 29.5, 29.4, 29.3, 27.1, 26.8, 22.7, 21.7, 19.6, and 14.1; Anal. Calcd. for $C_{28}H_{42}N_4O_2$ (F.W. = 466.66): C, 72.07; H, 9.07; N, 12.01; and O, 6.86;. Found: C, 71.78; H, 10.27; and N, 11.86. MALDI-TOF MS (Fig. S22): Calcd. for $C_{28}H_{42}N_4O_2$ m/z 466.33 (100 %), 467.33 (31.8 %), 468.34 (5.0 %), Obtained as reduced form: 467.43 (63.2 %), 468.44 (100 %), 469.43 (67.1 %); FTIR (KBr pellet): ν (cm^{-1}) 3460, 2919, 2850, 1726, 1676, 1582, 1539; Raman (785 nm excitation, shift in cm^{-1}): 311, 425, 500, 608, 675, 741, 1231, 1351.

10-Eicosyl-7,8-dimethyl-10H-benzo[g]pteridine-2,4-dione (**FC20**): A mixture of **4** (0.792 g, 1.9 mmol), alloxan monohydrate (0.320 g, 2 mmol), and boric oxide (0.42 g, 6 mmol) in 60 mL glacial acetic acid was stirred at 60 °C for 6 h. A similar protocol used for synthesis of **FC8** was utilized. The TLC R_f of the target compound was 0.40 with 95:5 MC/MeOH. **FC20** was purified by flash chromatography on silica gel in 95:5 MC/MeOH to produce 0.491 g (0.94 mmol) of yellow crystals (yield: 50%); m.p.: 280 °C; ^{1}H NMR ($CDCl_3$ with TMS, 400 MHz, Figure. S3D) δ 8.36 (1H, s), 8.07 (1H, s), 7.39 (1H, s), 4.69 (2H, br s), 2.57 (3H, s), 2.46 (3H, s), 1.86 (2H, quint, J = 15.9 Hz), 1.52 (2H, quint, J = 15.5 Hz), 1.39 (2H, m), 1.25 (22H, s), and 0.88 (3H, t, J = 7.1 Hz); ^{13}C NMR ($CDCl_3$ with TMS, 100 MHz, Figure. S4D) δ 159.6, 155.2, 150.0, 148.3, 137.1, 136.1, 135.0, 132.8, 131.1, 115.3, 45.4, 31.9, 29.7, 29.7, 29.7, 29.7, 29.7, 29.7, 29.7, 29.7, 29.7, 29.7, 29.7, 29.7, 29.7, 29.7, 29.7, 29.7, 29.6, 29.5, 29.4, 29.3, 27.1, 26.8, 22.7, 21.7, 19.6, and 14.1; Anal. Calcd. for $C_{32}H_{50}N_4O_2$ (F.W. = 522.76): C, 73.52; H, 9.64; N, 10.72; and O, 6.12;. Found: C, 73.31; H, 10.64; and N, 10.94. MALDI-TOF MS: Calcd. for $C_{32}H_{50}N_4O_2$ m/z 522.39 (100

%), 523.40 (35.3 %), 524.40 (6.4 %), Obtained as reduced form: 523.49 (52.3 %), 524.50 (100 %), 525.5 (45.0 %); FTIR (KBr pellet): ν (cm^{-1}) 3376, 2920, 2849, 1723, 1672, 1582, 1546; Raman (785 nm excitation, shift in cm^{-1}): 298, 500, 608, 675, 741, 1232, 1352.

$\Phi_{R, (10, 8)}$ determination:

According to previous literature [1–3], the fluorescence quantum yield of a sample (Φ_S) is defined as following:

$$\Phi_S = \Phi_{ref} \times \frac{I_s \times \alpha_{ref} \times n_s^2}{I_{ref} \times \alpha_s \times n_{ref}^2} \quad (1)$$

where, I is area-based fluorescence intensity, α is absorption, n is refractive index of medium, and s and ref denote sample and reference, respectively. Because the samples were prepared in the same solvent (*i.e.*, *p*-xylene), the relative quantum yield (Φ_R) per a particular chirality is as follows:

$$\Phi_{R,(n,m)} = C \times \frac{I_{(n,m)}}{\alpha_{(n,m)}} \quad (2)$$

where, C is a constant. The most abundant species in the PLE map, the (10,8) tube, exhibits e^S_{11} and e^S_{22} at 1520 nm and 885 nm, respectively. As shown in Figure. 4C, absorption consists of PL-emitting α_{885nm} and nonradiative β_{885nm}. Because the chirality distribution and absorbance are quite similar irrespective of flavin derivatives evident by the PLE maps, except for **FC8**, we assumed that $\alpha_{(10,8)}$ is constant. For $I_{(10,8)}$, a different β_{885nm} consisting of nonradiative SWNT bundle and CI was observed. Therefore, because not all excited light is used for exciting the (10,8) tube, $I_{(10,8)}$ must be replaced by normalized $I_{(10,8)}$ ($I_{(10,8),\ normalized}$) considering the loss by β_{885nm} against the value of $\alpha_{885nm}/(\alpha_{885nm} + \beta_{885nm})$, producing

$$I_{(10,8),normalized} = \frac{I_{(10,8)}}{\alpha_{885nm}/(\alpha_{885nm} + \beta_{885nm})} \quad (3)$$

$I_{(10,8)}$ can be obtained by deconvolution, as shown in Figure. 4D.

$$\Phi_{R,(n,m)} = C \times \frac{I_{(n,m),normalized}}{\alpha_{(n,m)}} \quad (4)$$

Although there is uncertainty for $\alpha_{(10,8)}$, this approach would provide a qualitative Φ_R for SWNT dispersion containing CI and bundled SWNT.

Geometric modeling for Flavin-wrapped SWNT:

Flavin exhibits a well-defined self-assembly motif on SWNT [4–6]. Flavin helix has the most stable assembly on the (8,6) tube. This motif is based on an 8_1 helix of flavin dimer, which is a flavin helix unit cell formed by aligned H-bonding dimer. The 8_1 flavin helix unit cell is formed by eight pairs of facing flavin dimers that are created by a 45º rotation and 3.13 Å translation along the longitudinal direction around a virtual circle 1.63 nm in diameter. Such helical flavin wrapping on SWNT has been verified experimentally [4,7,8] and theoretically [9], analogous to the schematic in Figure. S13A–S13B, although geometry-optimized flavin dimers deviated from longitudinal to 'chevron' shape to minimize free volume on SWNT while maintaining their quadruple H-bonding. This 'chevron' modification does not alter the occupied surface on SWNT.

The SWNT helical motif with different d_t varies [6]. For instance, the d_t of tubes (6,5) and (8,6) is 0.75 and 0.95 nm, respectively. Moreover, (6,5) and (8,6) tubes contain 7_1 and 8_1 helices with flavin dimers, respectively, suggesting that increasing d_t 0.2 nm results in two additional flavins in the unit cell. Therefore, (8,10) with a 1.22 nm d_t and 488 carbon atoms in a 3.33 nm translational (T) length is expected to accommodate 9_1 flavin helix consisting of 18 flavins. The flavin dimer was rotated by 40º, followed by a 2.78 Å translation along z-axis nine times in the 2.5 nm unit cell length to generate the 9_1 helical motifs.

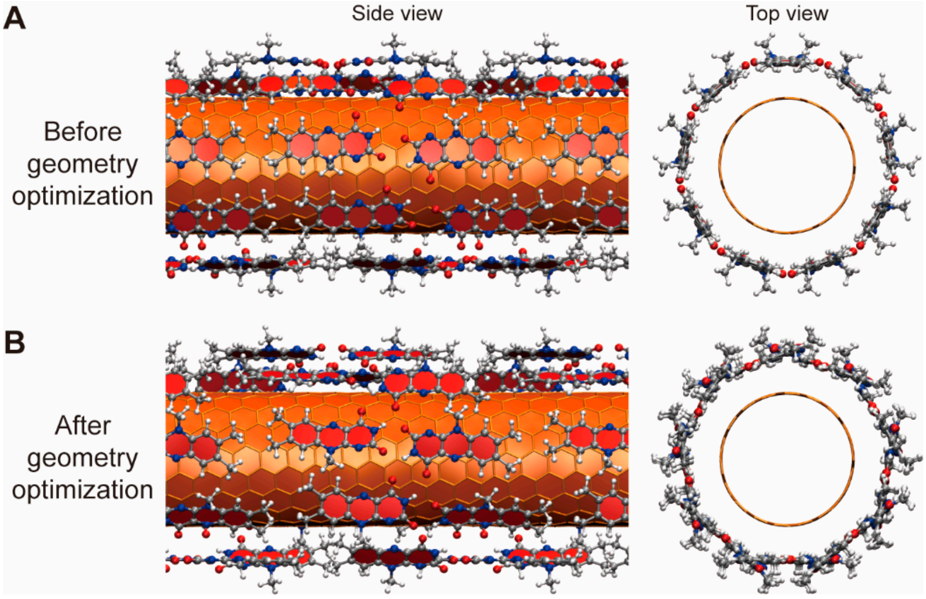

Scheme S1. Sideview (left) and topview (right) of 9_1 flavin helix-wrapped (8,10) tube (**A**) before and (**B**) after geometry optimization. Lumiflavin (methyl flavin) was utilized for molecular mechanics (MM) simulation.

The above figure is atomistic configurations of such 9_1 flavin helix-wrapped (8,10) SWNT before and after geometry optimization by MM simulation. Density functional method, higher level of theoretical calculation, is not utilized because of larger atom numbers (*i.e.*, 1464). After the optimization, the helical flavin wrapping is still maintained albeit the facing flavins are slightly tilted each other. Topview clearly shows such flavin tilt. Nevertheless, the result shows the 9_1 flavin helix can be formed on SWNT surface without structural distortion.

For the 9_1 flavin helix on (8,10) SWNT, the distance between adjacent flavin pairs along the SWNT d_t direction (*i.e.*, 0.66 nm) is similar to that of 8_1 flavin helix on (6,8) SWNT (*i.e.*, 0.64 nm). Therefore, the number of SWNT carbon atoms occupied by one flavin supramolecular helix composed of 18 flavin molecules is 366.7. The determination of dispersibility required for SWNT in terms of flavin side chain was calculated as follows. Set the number of carbon atoms in the flavin side chain to n_C. Then, dividing the number

of carbon side chains in 18 flavins of 9_1 helix (18 × n_C) by the number of underlying carbon atoms in SWNT (*i.e.*, 366.7) yields **0.049 × n_C**, which is a figure of merit for estimating the dispersibility to SWNT dispersion with average d_t = 1.22 nm. Considering the number of carbon atoms in the side chain, **FC12** exhibits a figure of merit of 0.59.

Smaller d_t SWNT, such as the (6,8) tube, possesses a d_t and longitudinal length of 0.95 nm and 2.59 nm, respectively, and contains 296 carbon atoms. (6,8) SWNT is wrapped by 8_1 FMN dimer motif (*i.e.*, formed from a partially-hydrogen bonded flavin dimer rotated 45º and translated by 3.13 Å along the *z*-axis eight times). The nominal unit length of the flavin supramolecular assembly on (6,8) chirality is 2.5 nm and contains 16 flavin molecules. Therefore, the number of SWNT carbon atoms occupied by one flavin supramolecular helix composed of 16 flavin molecules is 285.6. The following was used to calculate the dispersibility required for SWNT in terms of the flavin side chain. Then, dividing the number of carbon side chains in the 16 flavin unit supramolecule (16 × n_C) by the underlying carbon atoms in (6,8) SWNT (*i.e.*, 285.6) produces **0.056** × n_C.

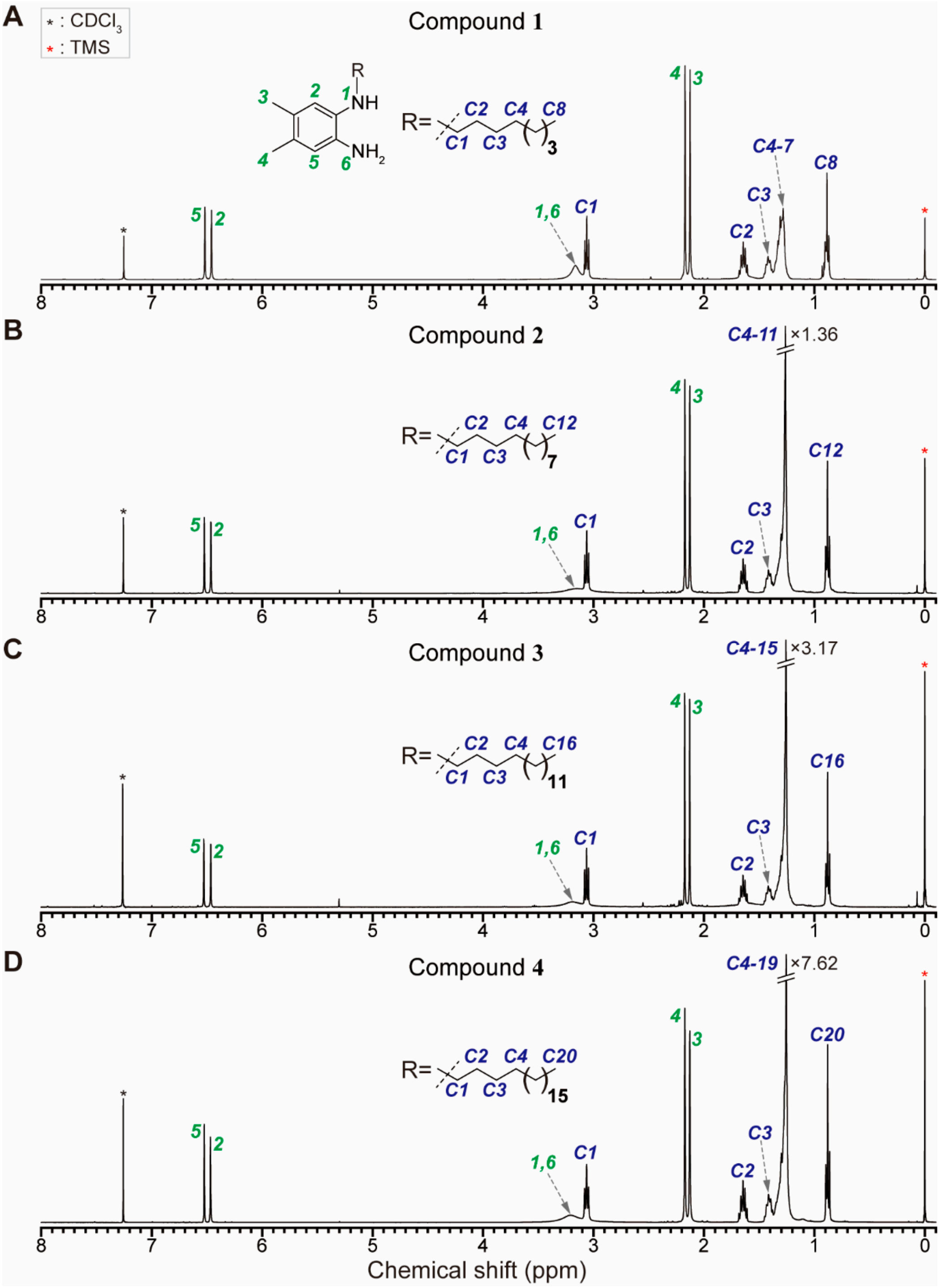

Figure S1. (**A–D**) ^{1}H NMR spectra of **1–4** in $CDCl_3$ acquired with Larmor frequency at 400 MHz. The asterisks indicate the peaks originating from solvents.

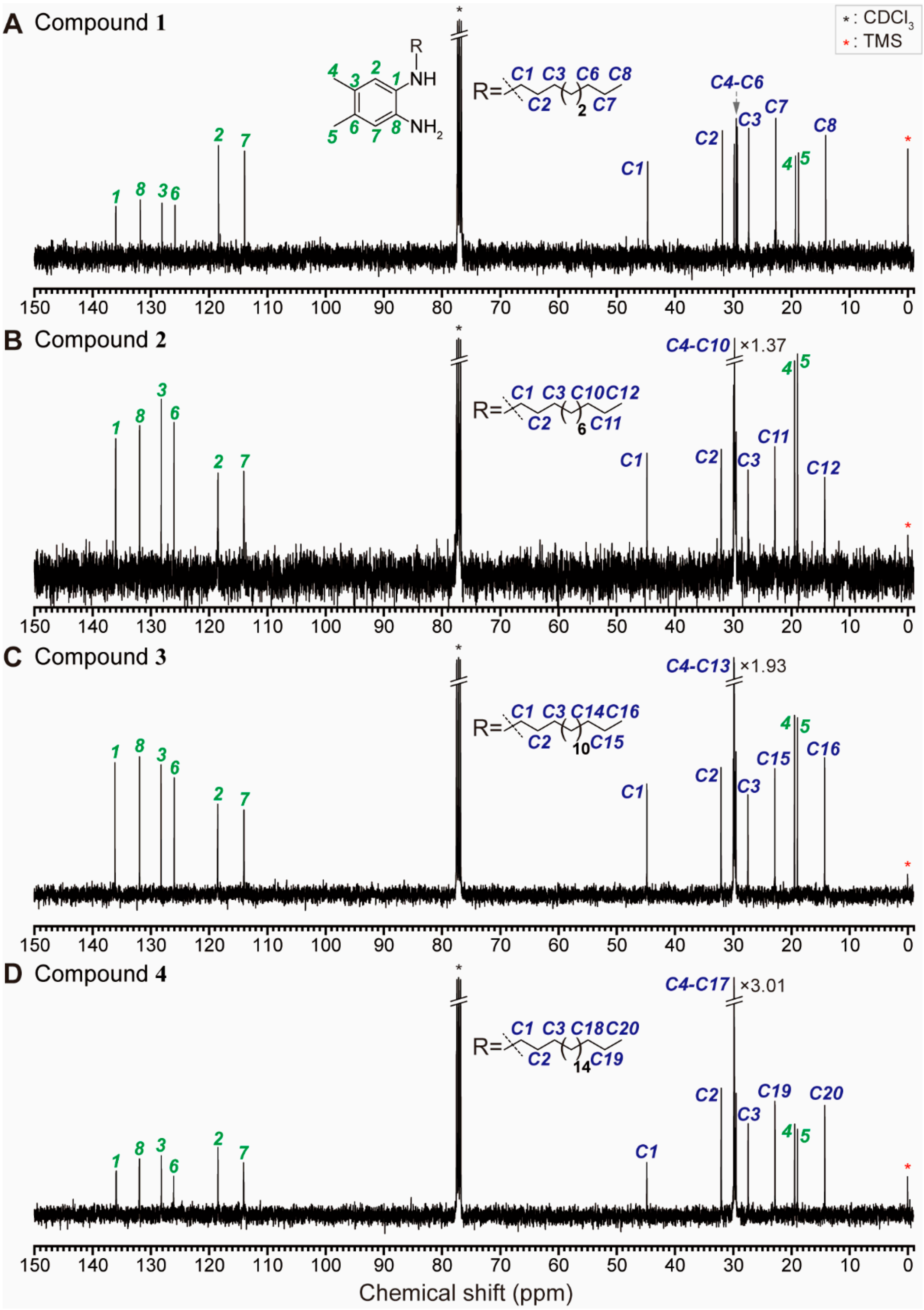

Figure S2. (**A**–**D**) ^{13}C NMR spectra of **1**–**4** in $CDCl_3$ acquired with Larmor frequency at 100 MHz. The asterisks indicate the peaks originating from solvents.

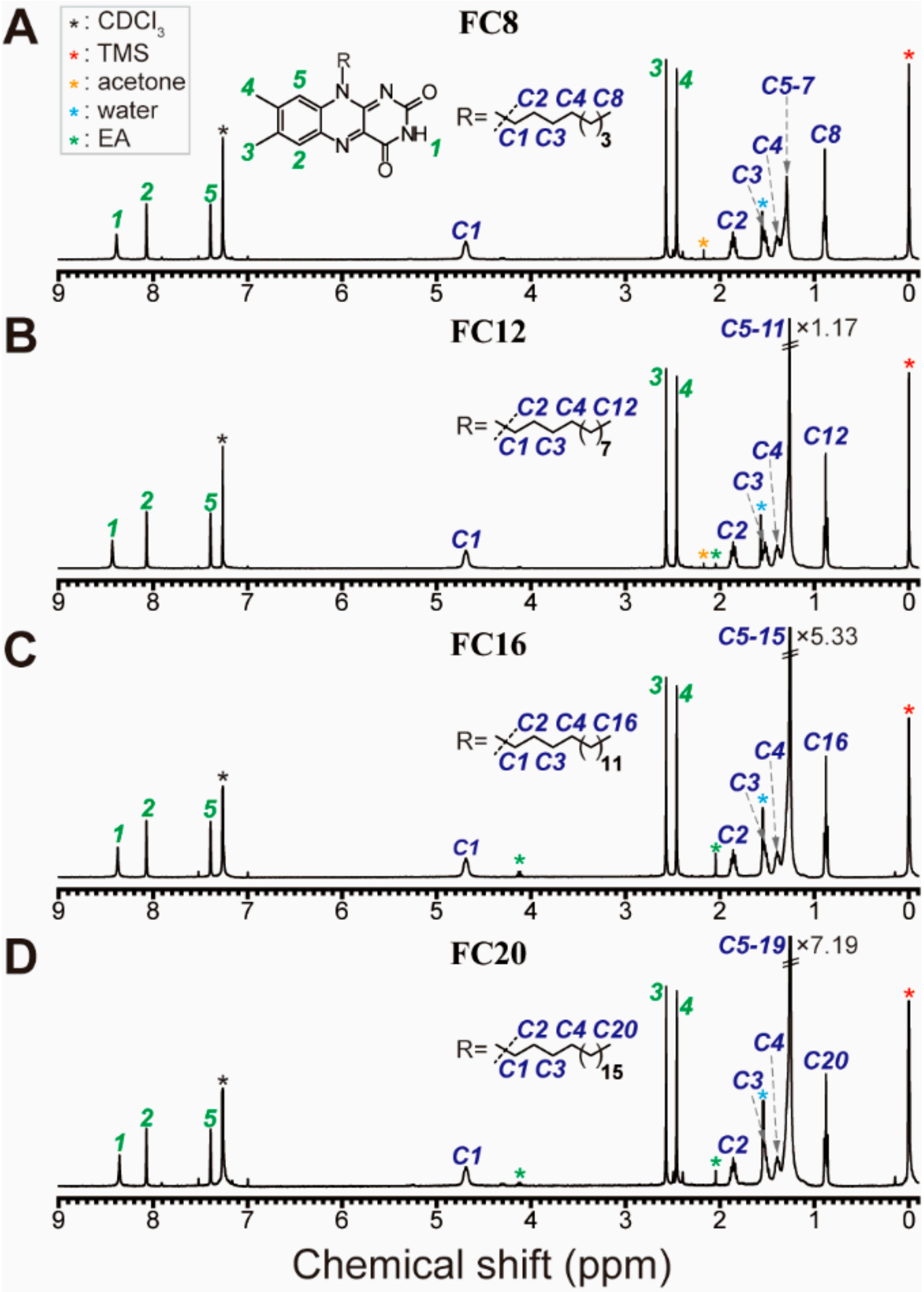

Figure S3.(A–D) ^{1}H NMR spectra of **FC8**, **FC12**, **FC16**, and **FC20** in $CDCl_3$ acquired at a Larmor frequency of 400 MHz. The asterisk indicates peaks originating from residual solvents.

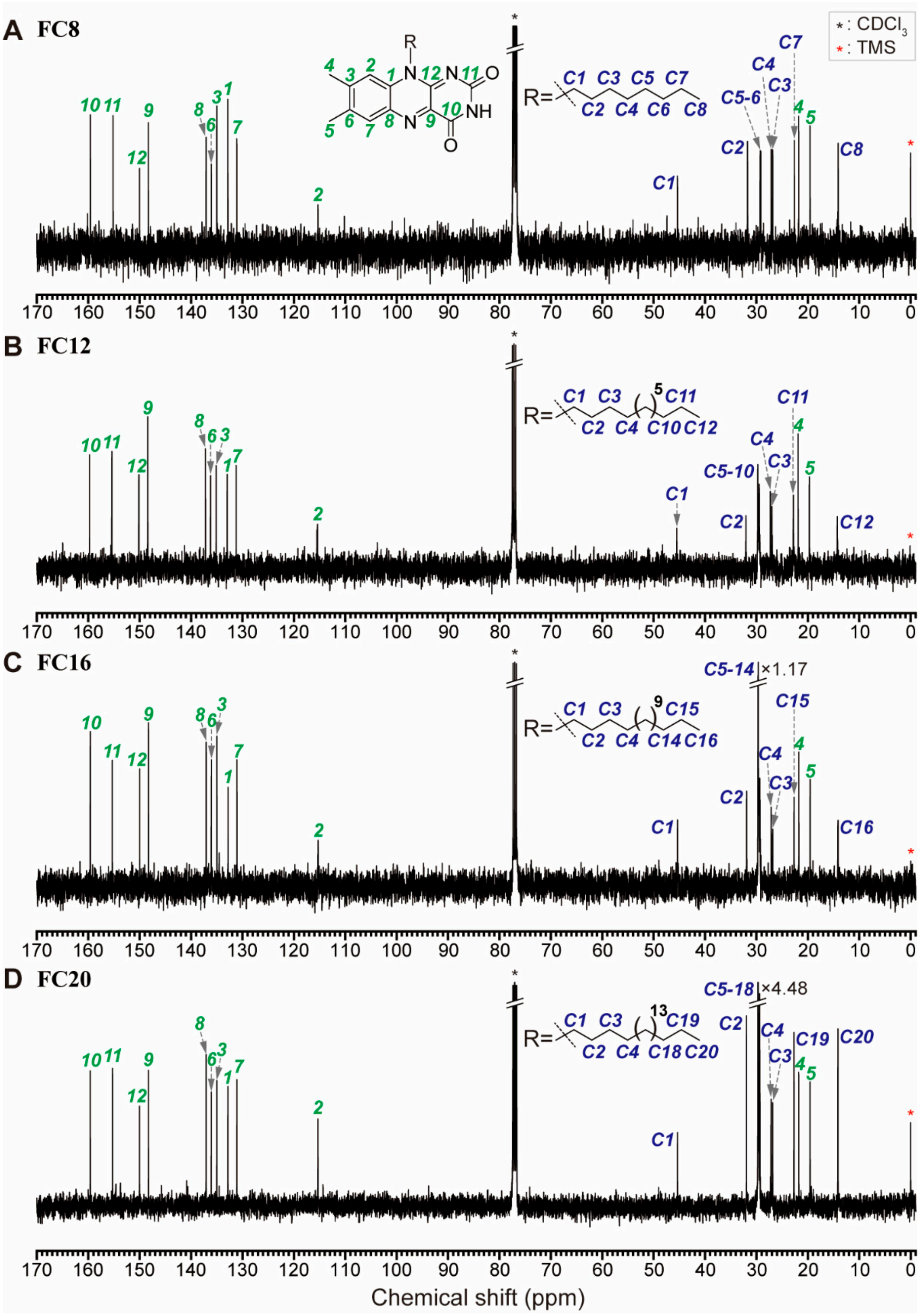

Figure S4. (**A–D**) ^{13}C NMR spectra of **FC8**, **FC12**, **FC16**, and **FC20** in $CDCl_3$ acquired with Larmor frequency at 100 MHz. The asterisks indicate the peaks originating from solvents.

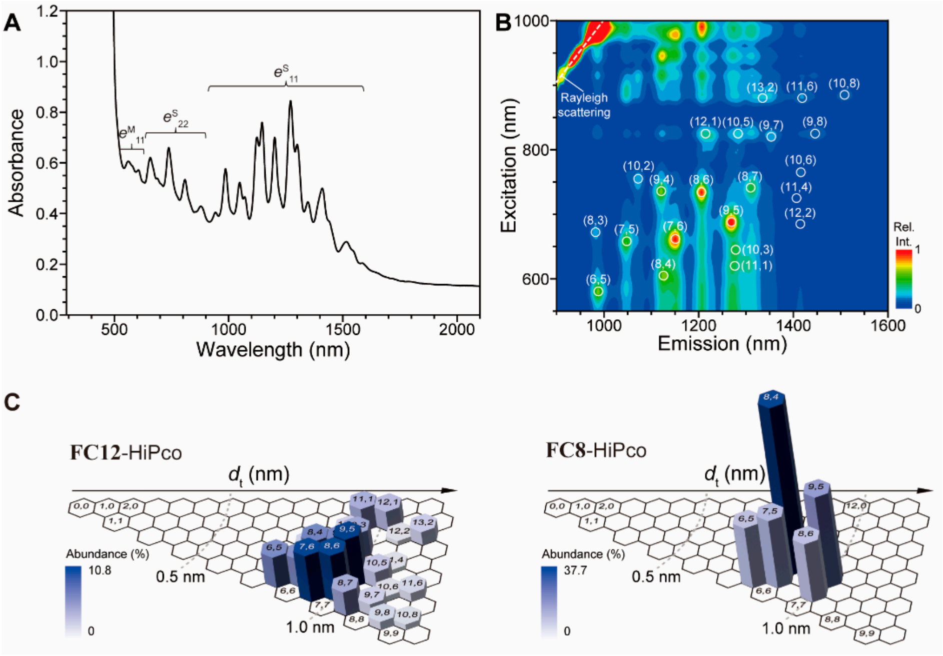

Figure S5. (**A**) Absorption spectrum and (**B)** corresponding PLE map of 2.44 mM **FC12**-HiPco dispersion centrifuged at 5 k*g*. (**C**) Weisman plots of PL-based (*n*, *m*) chirality abundance of **FC12**- and (**B**) **FC8**-HiPco.

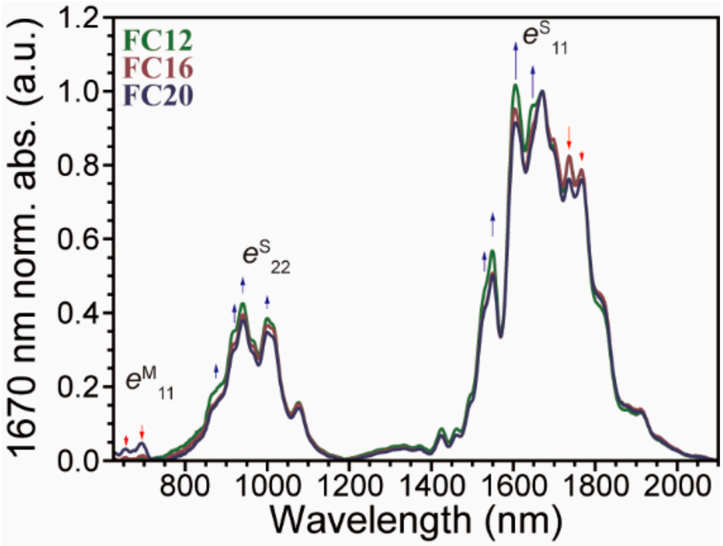

Figure S6. Larger d_t SWNT propensity of flavin with longer l_s. Background-subtracted absorption spectra of **FC12**-, **FC16**-, and **FC20**-PSWNT dispersions with 5 k*g* centrifugation. These spectra were normalized by absorbance at 1670 nm. Blue and red arrows denote the absorption increasing and decreasing, respectively.

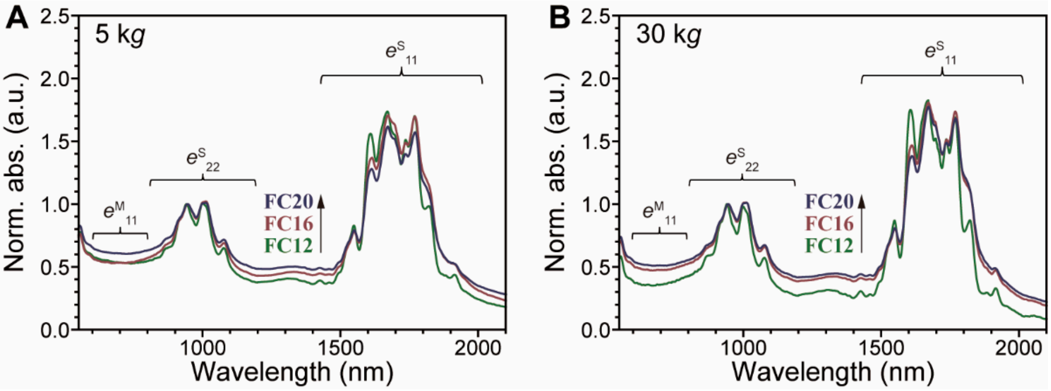

Figure S7. Dispersion of PSWNT with low flavin concentration (0.5 mM). (**A**) UV-vis-SWIR absorption spectra of PSWNT dispersed by **FC12**, **FC16,** and **FC20** centrifugated at (**A**) 5 k*g* and (**B**) 30 k*g*.

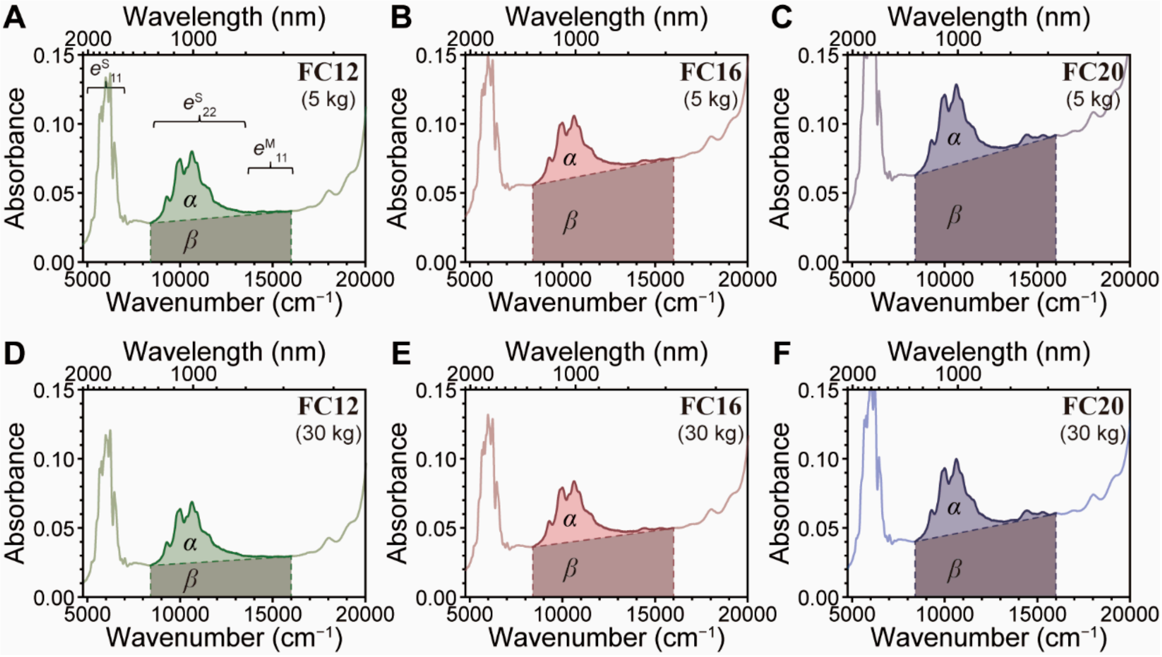

Figure S8. (**A**–**F**) Defining α and β in the absorption spectra in wavenumber scale.

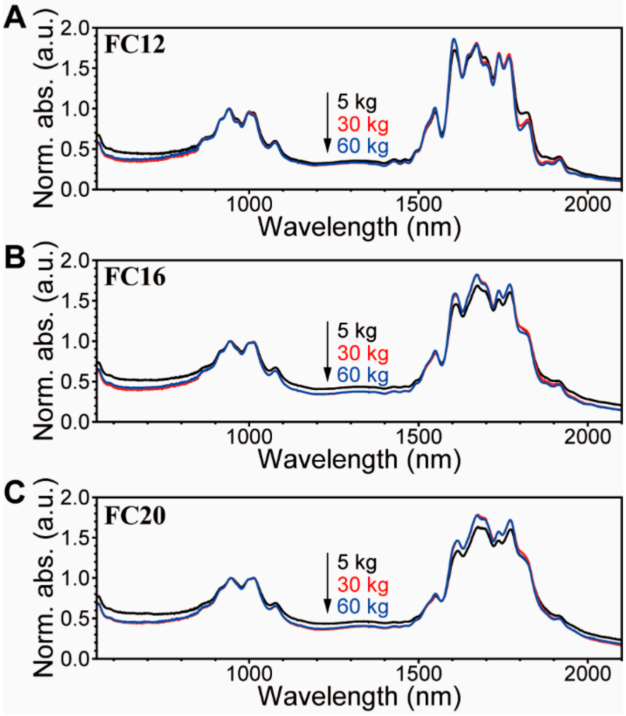

Figure S9. Comparison of PSWNT dispersions according to centrifugal forces. (A) UV-vis-SWIR absorption spectra of PSWNT dispersed by (**A**) **FC12**, (**B**) **FC16,** and (**C**) **FC20** with different centrifugal forces.

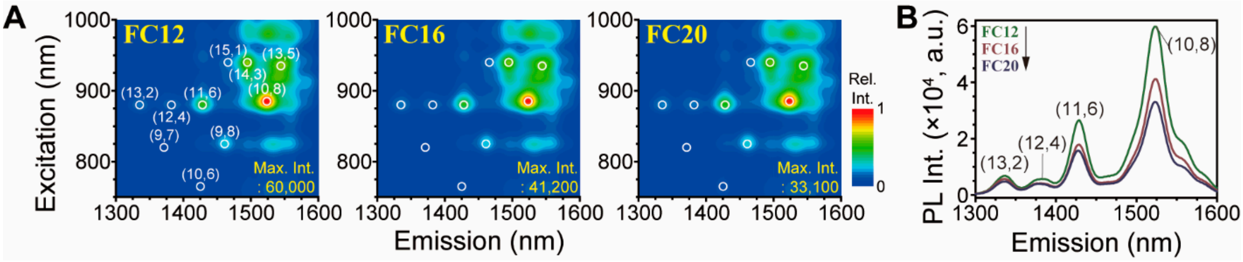

Figure S10. (**A**) PLE maps of **FC12**-, **FC16**-, and **FC20**-PSWNT dispersions centrifuged at 30 kg, respectively. (**B**) Overlaid PL emission spectra of PSWNT according to the *l*s. Excitation: 885 nm.

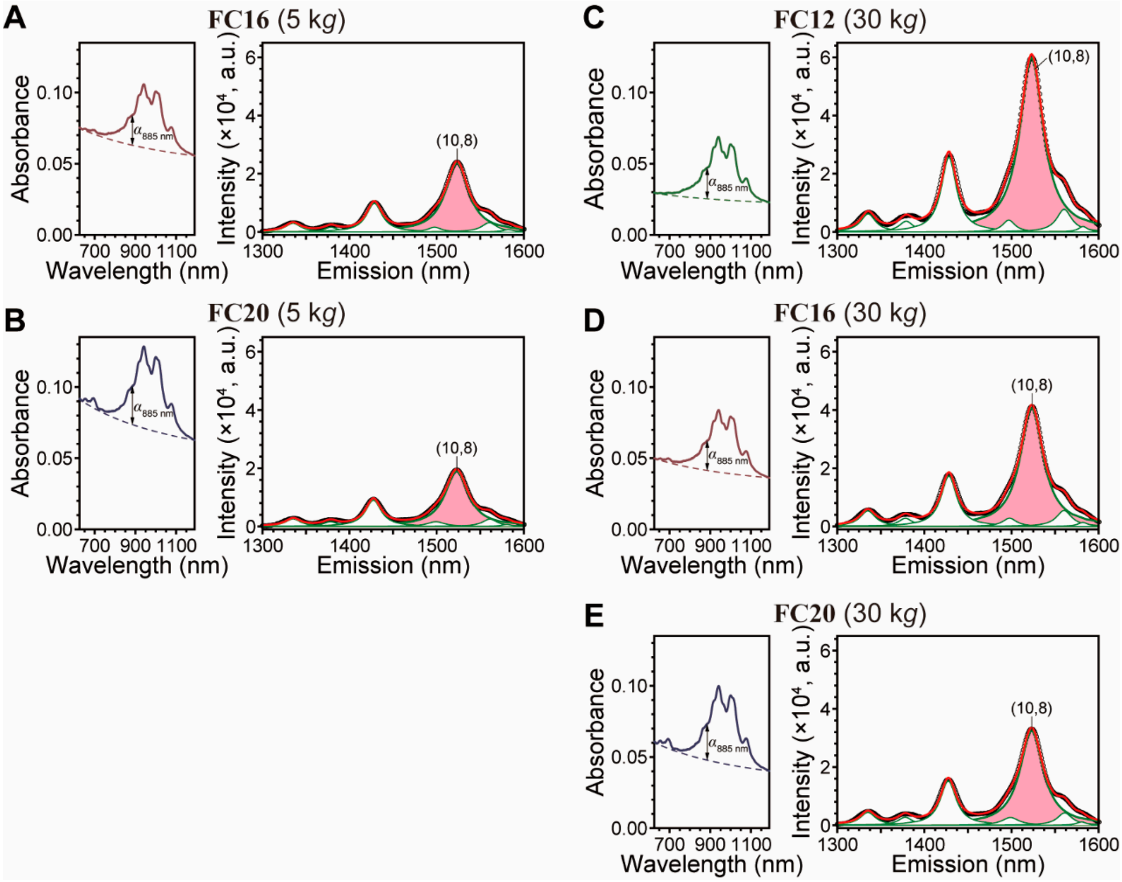

Figure S11. Comparison of absorption (left) and PL emission (right) spectra for $\Phi_{R,\ (10,8)}$ determinations from **FC12**-, **FC16**-, and **FC20**-PSWNT dispersions with (**A**–**B**) 5 kg and (**C**–**E**) 30 kg centrifugations. (Left) Absorption spectrum with interface (dashed line) between α_{885nm}) and β_{885nm} and (right) pink-shaded area denotes the integrated area from PL emission of (10,8) SWNT. Each curve was deconvoluted by Lorentzian fit.

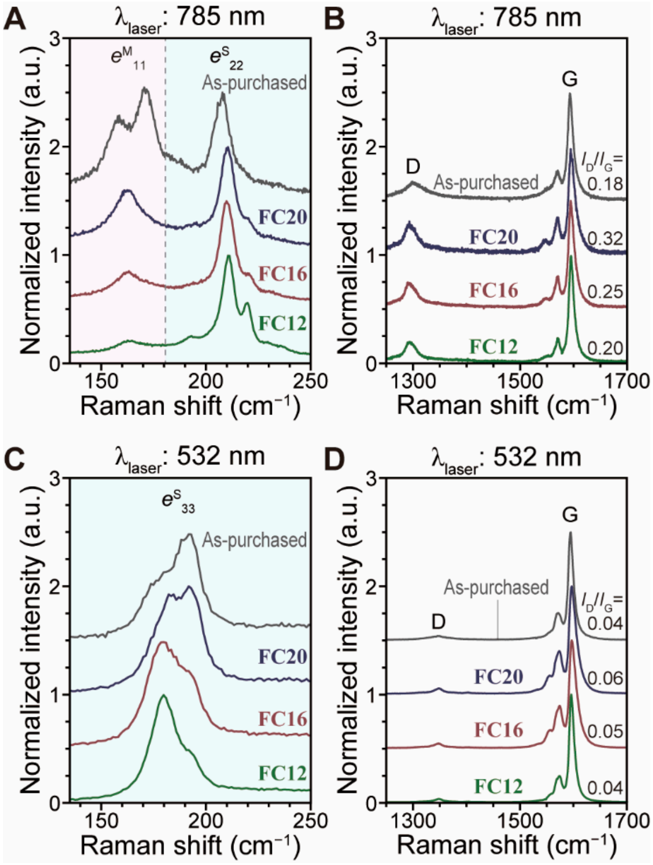

Figure S12. Raman spectra change of PSWNT with 30 k*g* centrifugation with varying flavin *l*s. (**A**) RBM and (**B**) G band spectra excited by 785 nm. As-purchased PSWNT was used as a control. The dispersions were deposited on 285 nm-thick SiO_2/Si substrate and were washed with copious amount of acetone to remove flavins. (**C**) RBM and (**D**) G band spectra excited by 532 nm whose samples were prepared in a similar manner.

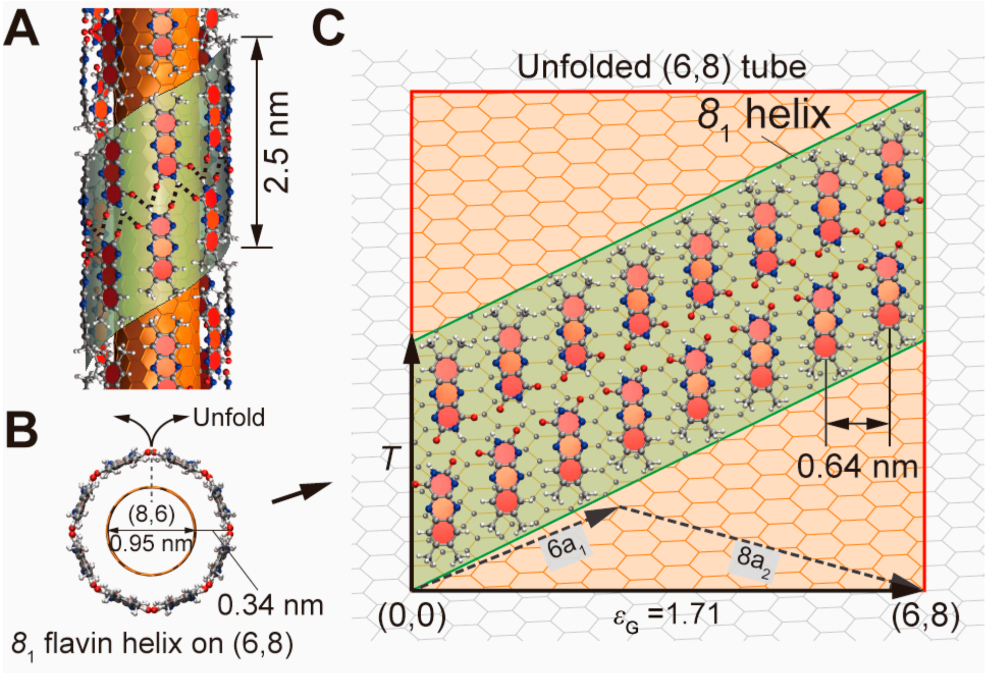

Figure S13. Calculation of the l_S per carbon atom in the SWNT using the geometry of 8_1 flavin helix-wrapped (6,8) tube. (**A**) Side- and (**B**) top-views of helical flavin wrapping spaced with vdW distance (0.34 nm) on (6,8) tube with d_t = 0.95 nm. Note that flavin unit length is 2.5 nm. (**C**) Projected view of 16 isoalloxazine rings on stretched graphene sheet with ε_G = 1.71 while the length of translational vector (T) of (6,8) tube remains unchanged.

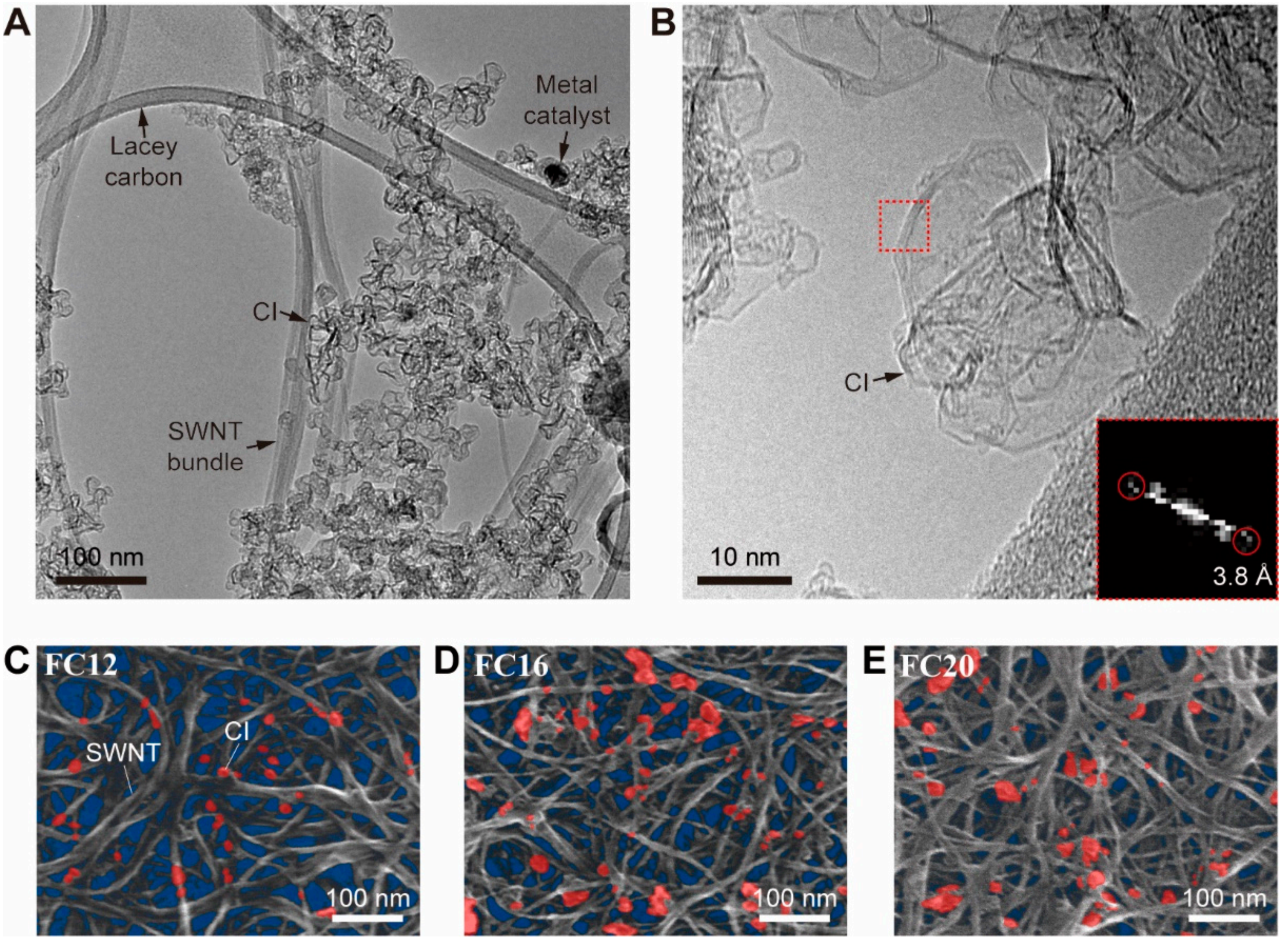

Figure S14. Variation of CI present in PSWNT films prepared by varying flavin *l*s. (**A**) TEM image of as-purchased PSWNT containing SWNT, CIs, and metal catalyst dispersed by NMP. (**B**) High-magnification TEM image of CI and its FFT image as inset. SEM images of PSWNT films which are prepared by (**C**) **FC12**-, (**D**) **FC16**-, and (**E**) **FC20**-PSWNT dispersions. CI and background are marked by red and blue for visual comparison.

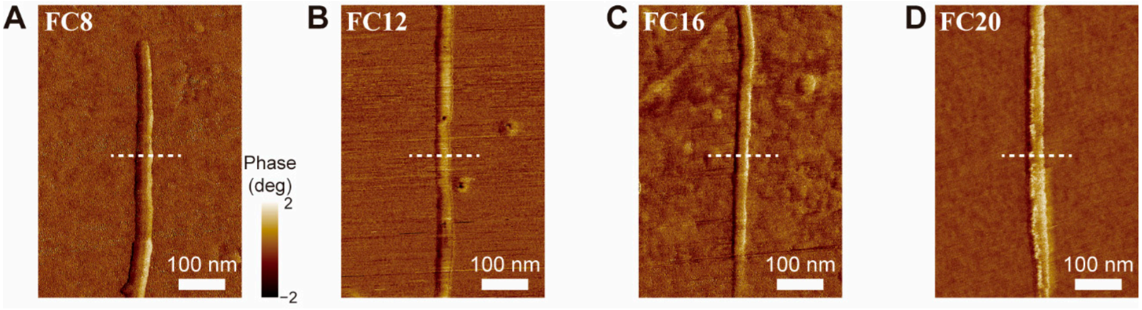

Figure S15. The corresponding AFM phase images of (**A**) **FC8**-, (**B**) **FC12**-, (**C**) **FC16**-, and (**D**) **FC20**-wrapped PSWNT of Figures 7A to 7D. Dashed lines indicate the lines for Figure 7E.

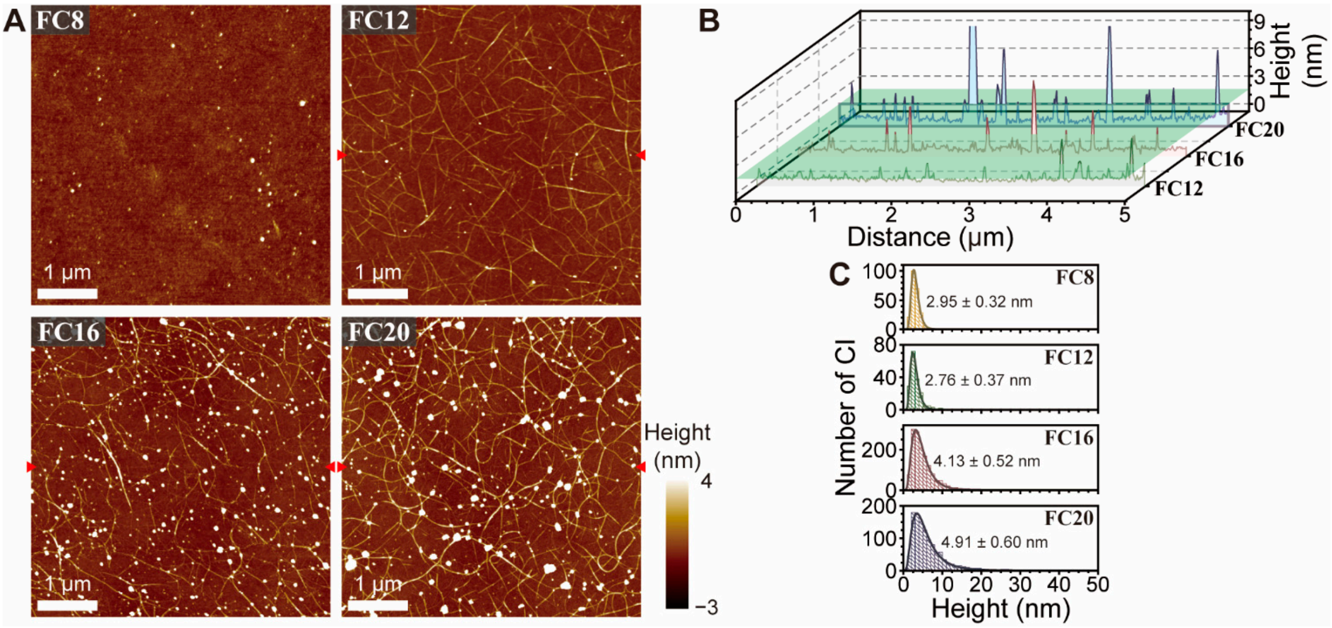

Figure S16. SWNT and CI morphology trends in **FC8**-, **FC12**-, **FC16**-, and **FC20**-PSWNT dispersions centrifuged at 30 k*g*. (**A**) AFM height images of each dispersion deposited on 285 nm-thick SiO_2/Si substrate from 30 k*g* centrifugation. (**B**) The representative height profiles whose position were indicated by pair of triangles in the AFM height images. Limit of individualized PSWNT is indicated by 1.6 nm green plane. (**C**) Height histograms of CI fitted by using a Lognormal function. Average height and standard deviation are listed.

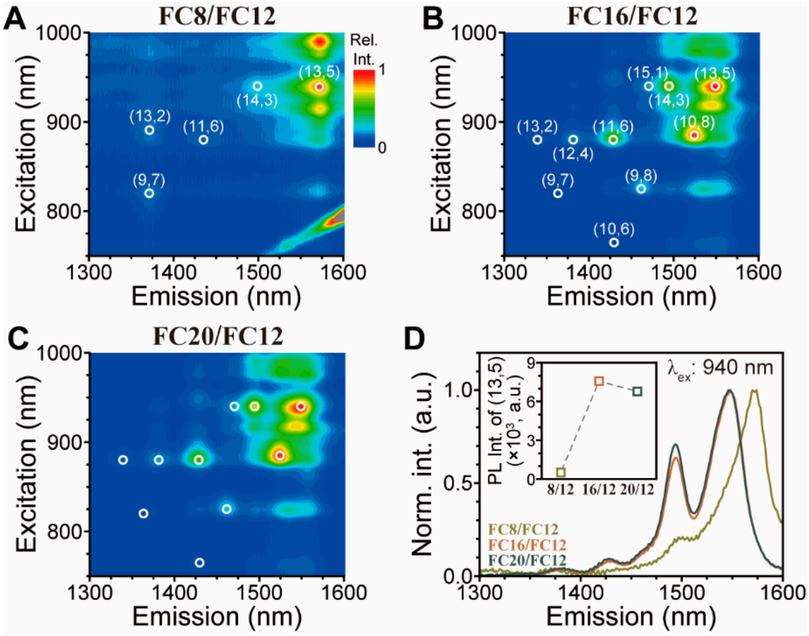

Figure S17. PLE maps of PSWNT dispersions by using tandem flavin surfactants (**A**) **FC8/FC12**, (**B**) **FC16/FC12,** and (**C**) **FC20/FC12**. (**D**) The corresponding PL emission spectra excited by 940 nm. The spectra were normalized against the maximum intensity of (13,5). Inset: PL intensity change of (13,5) SWNT according to combinations of flavin derivatives.

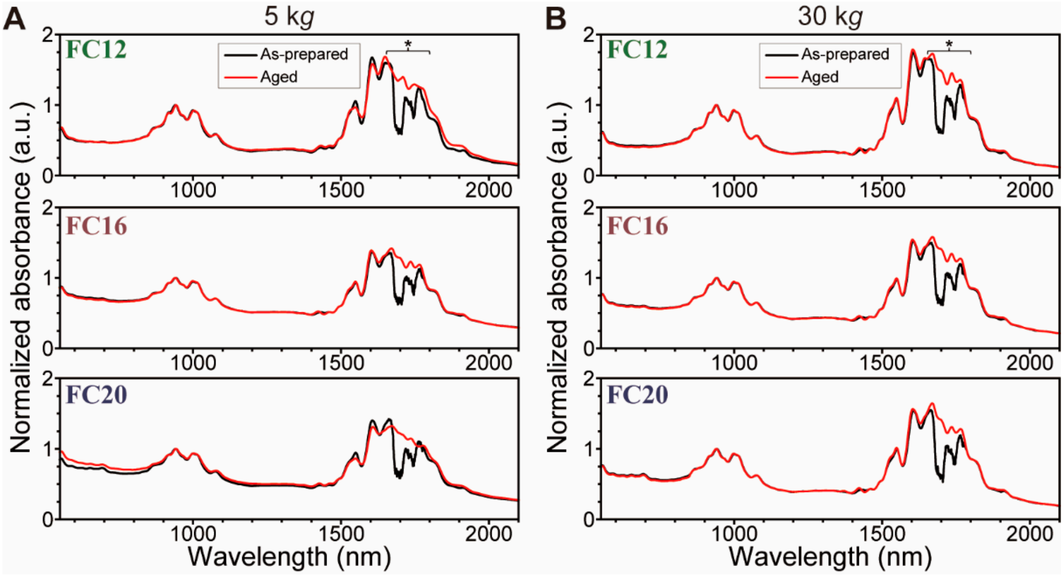

Figure S18. Comparison of absorption spectra from the as-prepared (black) and the aged (red) samples of **FC12**-, **FC16**-, and **FC20**-PSWNT dispersions after (**A**) 5 k*g* and (**B**) 30 k*g* centrifugations. The asterisks indicate residual *p*-xylene absorption.

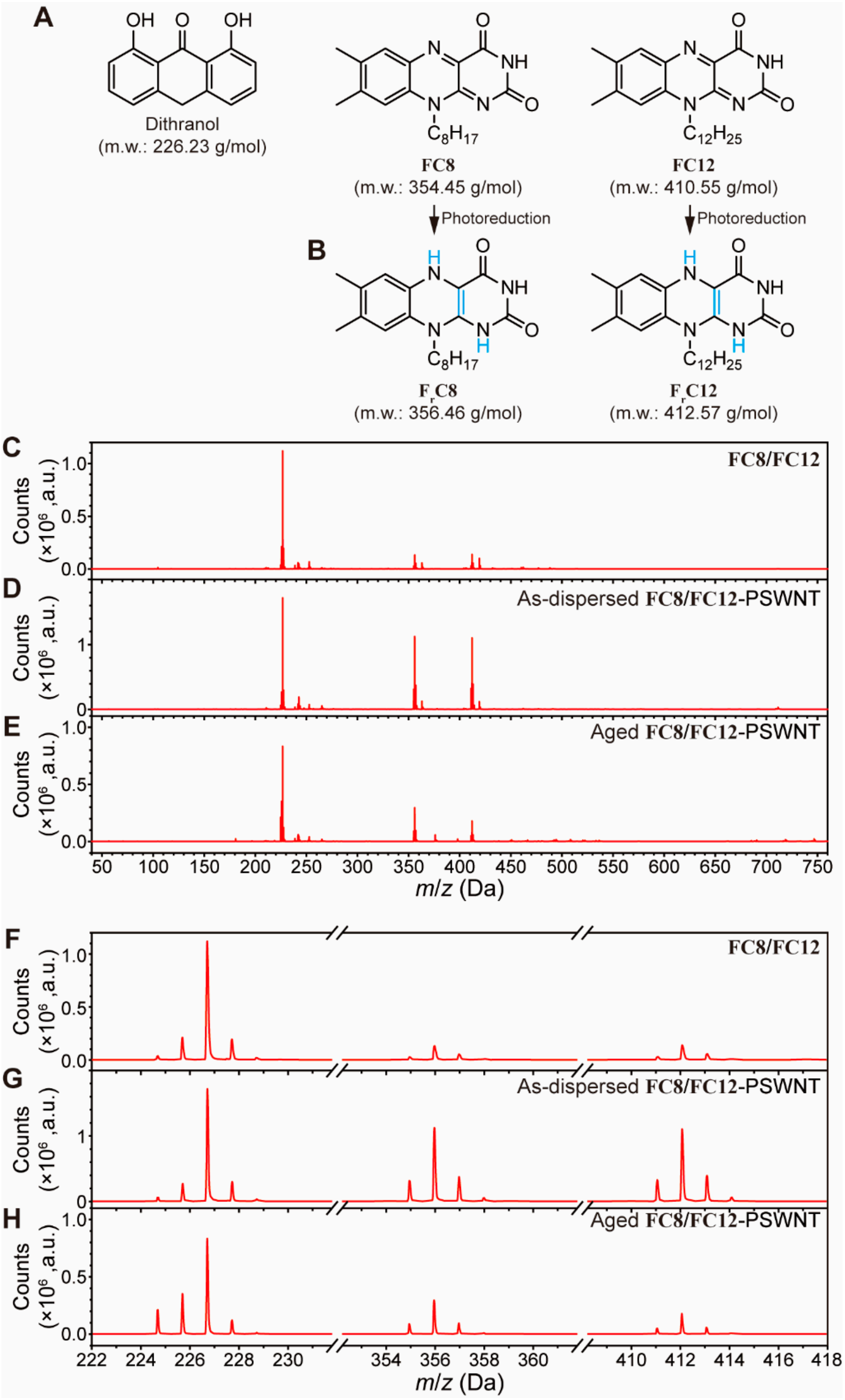

Figure S19. Chemical structures in MS spectra. (**A**) Chemical structures of dithranol as a reference, **FC8** and **FC12**. (**B**) Chemical structure of laser-induced reduced forms **F$_r$C8** and **F$_r$C12**. MALDI-TOF MS spectra of (**C**) isomolar **FC8/FC12** dispersion, (**D**) the as-prepared **FC8/FC12**-PSWNT, and (**E**) the aged **FC8/FC12**-PSWNT. (F to G) Corresponding zoom-in MS spectra.

A

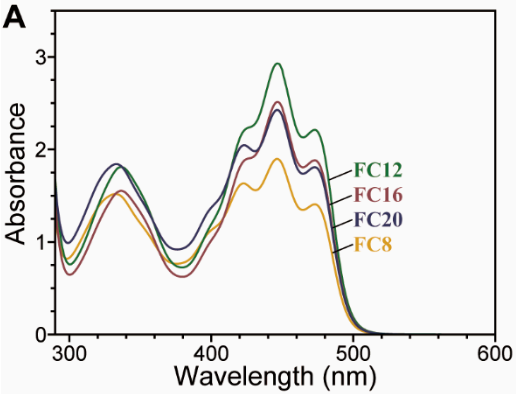

B

	Abs.	λ_{max} (nm)	Solubility (mg/mL)	Solubility (mM)
**FC8**	1.90	446	0.53	1.51
**FC12**	2.93	447	0.95	2.32
**FC16**	2.51	447	0.93	1.99
**FC20**	2.43	446	1.01	1.93

Figure S20. Solubility determination of flavin derivatives by using absorption spectra. (**A**) Absorption spectra with 1 mm beam-path cuvette and (**B**) solubilities of **FC8**, **FC12**, **FC16,** and **FC20** in *p*-xylene.

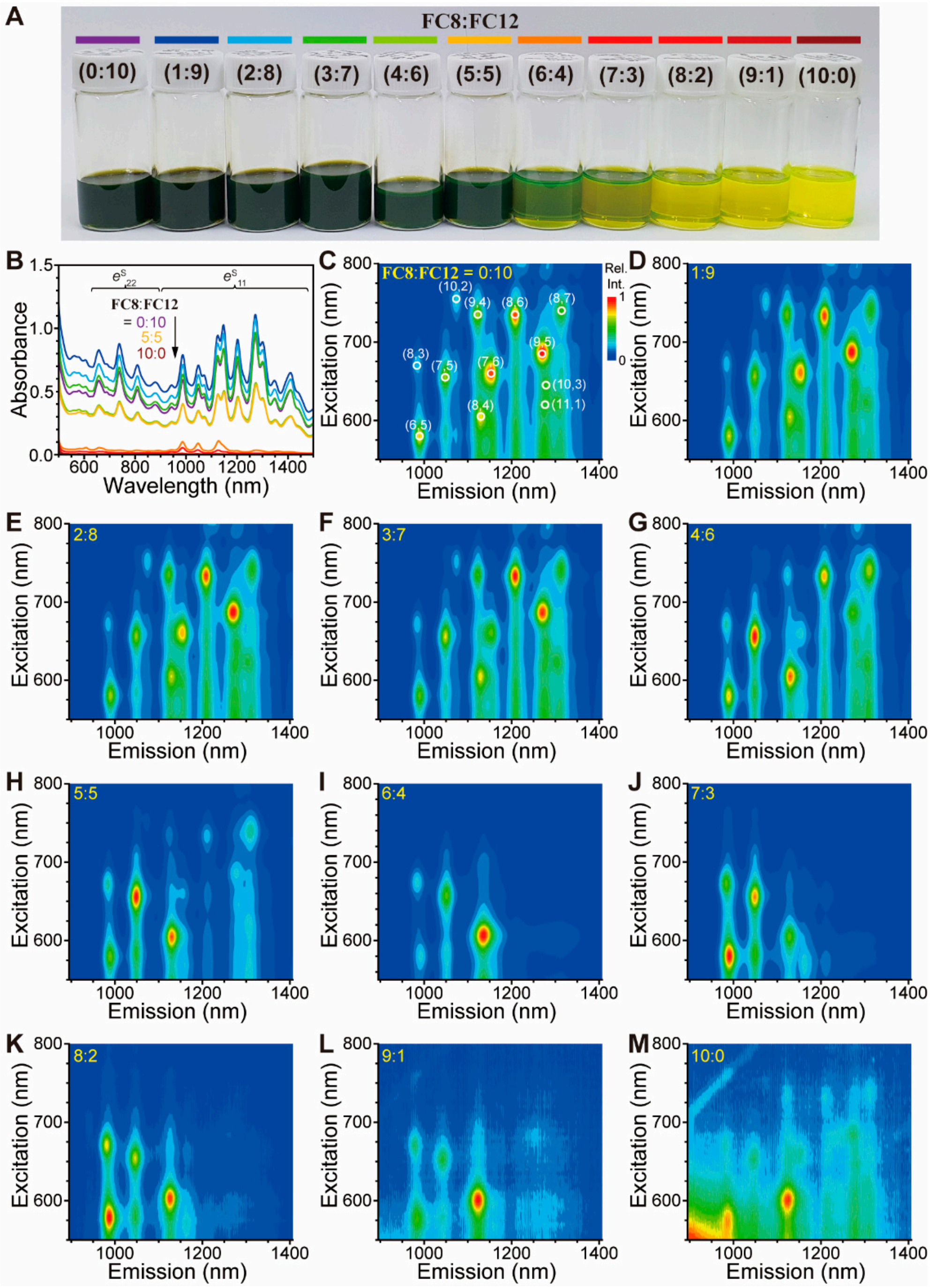

Figure S21. Effect of **FC8/FC12** ratio on d_t distribution of HiPco dispersion. (**A**) Photograph and (**B**) absorption spectra of **FC8/FC12**-HiPco dispersion with varying **FC8/FC12** ratio. Color variation from purple to red denotes the change of **FC8/FC12** ratio in HiPco dispersion. (**C**–**M**) PLE maps of HiPco dispersion with varying **FC8/FC12** ratio. Samples with greater absorption (*i.e.*, 0:10 to 5:5 ratio) were diluted to eliminate fluorescence reabsorption.

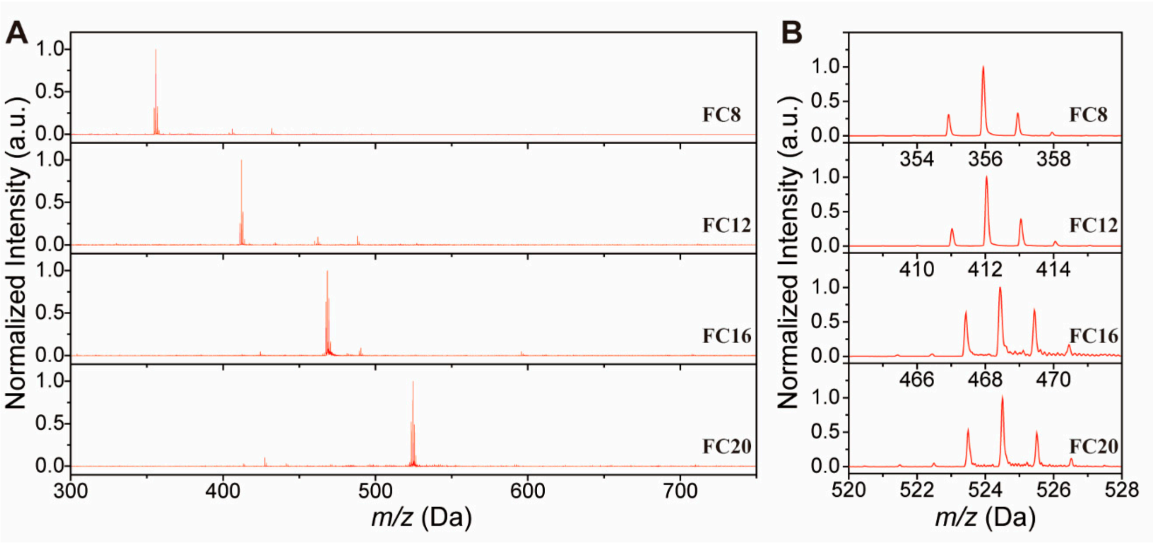

Figure S22. (**A**) Entire and (**B**) compound spectra of **FrC8**, **FrC12**, **FrC16**, and **FrC20** acquired by MALDI-TOF MS.

Table S1. δ and its subcomponents (δ_D, δ_P, and δ_H) of flavin side chains, *p*-xylene, and representative nano carbon allotropes.

Entry		δ_D ($MPa^{1/2}$)	δ_P ($MPa^{1/2}$)	δ_H ($MPa^{1/2}$)	δ ($MPa^{1/2}$)	$\Delta\delta$ against *p*-xylene ($MPa^{1/2}$)	Ref.
**Solvent**	*p*-Xylene	16.6	1	1	17.0		[10]
**Side chain of flavin**	*n*-Octyl	**15.8**	**0**	**0**	**15.8**	**1.6**	
	n-Dodecyl	16.1	0	0	16.1	1.5	
	n-Hexadecyl	16.3	0	0	16.3	1.4	
	n-Eicosyl	16.4	0	0	16.4	1.4	
**Nano carbon Isotopes**	CNO	15.9	10	8	20.4	11.4	[11]
	SWNT	17.8	7.5	7.6	20.8	9.3	[12]
	Graphene	18.0	9.3	7.7	21.7	10.8	[13]
	CB	17.2	8.5	11.6	22.4	13.0	[14]

References

1. Ju, S.-Y.; Kopcha, W.P.; Papadimitrakopoulos, F. Brightly Fluorescent Single-Walled Carbon Nanotubes *via* an Oxygen-Excluding Surfactant Organization. *Science* **2009**, *323*, 1319-1323.
2. Crochet, J.; Clemens, M.; Hertel, T. Quantum Yield Heterogeneities of Aqueous Single-Wall Carbon Nanotube Suspensions. *J. Am. Chem. Soc.* **2007**, *129*, 8058-8059.
3. Choi, I.-S.; Park, M.; Koo, E.; Ju, S.-Y. Dispersions of Carbon Nanotubes by Helical Flavin Surfactants: Solvent Induced Stability and Chirality Enrichment, and Solvatochromism. *Carbon* **2021**, *184*, 346-356.
4. Ju, S.-Y.; Doll, J.; Sharma, I.; Papadimitrakopoulos, F. Selection of Carbon Nanotubes with Specific Chiralities Using Helical Assemblies of Flavin Mononucleotide. *Nat. Nanotechnol.* **2008**, *3*, 356-362.
5. Konevtsova, O.V.; Roshal, D.S.; Dmitriev, V.P.; Rochal, S.B. Carbon Nanotube Sorting due to Commensurate Molecular Wrapping. *Nanoscale* **2020**, *12*, 15725-15735.
6. Sharifi, R.; Samaraweera, M.; Gascón, J.A.; Papadimitrakopoulos, F. Thermodynamics of the Quasi-Epitaxial Flavin Assembly around Various-Chirality Carbon Nanotubes. *J. Am. Chem. Soc.* **2014**, *136*, 7452-7463.
7. Sim, J.; Oh, H.; Koo, E.; Ju, S.-Y. Effect of Tight Flavin Mononucleotide Wrapping and Its Binding Affinity on Carbon Nanotube Covalent Reactivities. *Phys. Chem. Chem. Phys.* **2013**, *15*, 19169-19179.
8. Nakashima, N.; Fukuzawa, M.; Nishimura, K.; Fujigaya, T.; Kato, Y.; Staykov, A. Supramolecular Chemistry-Based One-Pot High-Efficiency Separation of Solubilizer-Free Pure Semiconducting Single-Walled Carbon Nanotubes: Molecular Strategy and Mechanism. *J. Am. Chem. Soc.* **2020**, *142*, 11847-11856.
9. Ogunro, O.O.; Wang, X.-Q. Quantum Electronic Stability in Selective Enrichment of Carbon Nanotubes. *Nano Lett.* **2009**, *9*, 1034-1038.
10. Krevelen, D.W.V. *Properties of Polymers*, 3rd ed.; Elsevier: New York, USA, **1990**; pp. 203.
11. Zuaznabar-Gardona, J.C.; Fragoso, A. Determination of the Hansen Solubility Parameters of Carbon Nano-Onions and Prediction of Their Dispersibility in Organic Solvents. *J. Mol. Liq.* **2019**, *294*, 111646.
12. Bergin, S.D.; Sun, Z.; Rickard, D.; Streich, P.V.; Hamilton, J.P.; Coleman, J.N. Multicomponent Solubility Parameters for Single-Walled Carbon Nanotube–Solvent Mixtures. *ACS Nano* **2009**, *3*, 2340-2350.
13. Hernandez, Y.; Lotya, M.; Rickard, D.; Bergin, S.D.; Coleman, J.N. Measurement of Multicomponent Solubility Parameters for Graphene Facilitates Solvent Discovery. *Langmuir* **2010**, *26*, 3208-3213.
14. Süß, S.; Sobisch, T.; Peukert, W.; Lerche, D.; Segets, D. Determination of Hansen Parameters for Particles: A Standardized Routine based on Analytical Centrifugation. *Adv. Powder Technol.* **2018**, *29*, 1550-1561.
